# Tandem Mass Tagging-Based Quantitative Proteomics Analysis Reveals Damage to the Liver and Brain of *Hypophthalmichthys molitrix* Exposed to Acute Hypoxia and Reoxygenation

**DOI:** 10.3390/antiox11030589

**Published:** 2022-03-19

**Authors:** Xiaohui Li, Cui Feng, Hang Sha, Tong Zhou, Guiwei Zou, Hongwei Liang

**Affiliations:** 1 Yangtze River Fisheries Research Institute, Chinese Academy of Fisheries, Wuhan 430223, China; lixiaohui@yfi.ac.cn (X.L.); fengcui@yfi.ac.cn (C.F.); sh1812@yfi.ac.cn (H.S.); zhoutong@yfi.ac.cn (T.Z.); 2 State Key Laboratory of Developmental Biology of Freshwater Fish, College of Life Sciences, Hunan Normal University, Changsha 410081, China; 3 Hubei Hongshan Laboratory, Huazhong Agricultural University, Wuhan 430070, China

**Keywords:** TMT, oxygen starvation, ferroptosis, redox reaction, *Hypophthalmichthys molitrix*

## Abstract

Aquaculture environments frequently experience hypoxia and subsequent reoxygenation conditions, which have significant effects on hypoxia-sensitive fish populations. In this study, hepatic biochemical activity indices in serum and the content of major neurotransmitters in the brain were altered markedly after acute hypoxia and reoxygenation exposure in silver carp (*Hypophthalmichthys molitrix*). Proteomics analysis of the liver showed that a number of immune-related and cytoskeletal organization-related proteins were downregulated, the ferroptosis pathway was activated, and several antioxidant molecules and detoxifying enzymes were upregulated. Proteomics analysis of the brain showed that somatostatin-1A (SST1A) was upregulated, dopamine-degrading enzyme catechol O methyltransferase (COMT) and ferritin, heavy subunit (FerH) were downregulated, and the levels of proteins involved in the nervous system were changed in different ways. In conclusion, these findings highlight that hypoxia–reoxygenation has potential adverse effects on growth, locomotion, immunity, and reproduction of silver carp, and represents a serious threat to liver and brain function, possibly via ferroptosis, oxidative stress, and cytoskeleton destruction in the liver, and abnormal expression of susceptibility genes for neurodegenerative disorders in the brain. Our present findings provide clues to the mechanisms of hypoxia and reoxygenation damage in the brain and liver of hypoxia-sensitive fish. They could also be used to develop methods to reduce hypoxia or reoxygenation injury to fish.

## 1. Introduction

In contrast to terrestrial ecosystems, the dissolved oxygen (DO) of aquatic ecosystems exhibits a wide range of temporal and spatial variations depending on biological and abiotic factors [1]. Hypoxia occurs when organisms (e.g., bacteria, plants, and animals) consume oxygen faster than they produce or acquire it. In aquatic environments with high biomass, respiration cycles and the photoperiod frequently result in hypoxia [2]. In particular, in midsummer, high peak temperatures following several days of cloudy weather contribute to acute overnight hypoxia [3]. Furthermore, in recent years, global water pollution and high-density fish farming have put tremendous pressure on aquatic systems and increased the risk of hypoxia [4]. Consequently, nighttime hypoxia and daytime reoxygenation occur frequently in aquatic environments, with associated negative effects on aquatic wildlife, including death [5].

Oxygen homeostasis is crucial for all aquatic organisms, and a complex series of neural and physiological modifications can be caused by large changes in environmental oxygen levels [6]. Hypoxic stress has powerful and direct effects on cellular function [7], inducing cytoskeleton disruption [8] and cellular apoptosis or necrosis in minutes [9]. Hypoxia-induced damage to the liver and brain cells of fish can cause death because of a lack of oxygen-dependent ATP generation in both organs (compared with that of the whole body, the brain’s metabolic rate is four-to-ten times higher and that of the liver is two times higher) [10]. Under ATP deficiency, cells cannot maintain ion pumping, resulting in cell depolarization [11], and most liver and brain cells in fish die due to apoptosis and necrosis [12]. In addition, studies have demonstrated that hypoxia could trigger encephalopathy, with a series of nervous and mental disorders as symptoms [13].

Post-hypoxia reoxygenation has been used traditionally to effectively ameliorate hypoxia-induced injury to fish. However, varying oxygen levels, from anoxia to hyperoxia, result in a correspondingly large variation in reactive oxygen species (ROS) production, which could aggravate tissue oxidative damage [14]. In mammals, ischemia–reperfusion (I/R) or hypoxia–reoxygenation (H/R) cause injury and have critical functions in certain clinical conditions, leading to hepatic oxidative damage, fibrosis, and ultimately death [15]. Cerebral I/R injury is a serious condition that leads to increased apoptosis of microglia and neurons in the brain [16]. Moreover, in vitro, hypoxia, reoxygenation, or both caused increasing apoptosis, which is the major cause of cell death [9]. However, investigations into the molecular mechanisms of hypoxia–reoxygenation injury in fish are lacking, particularly the effects on brain and liver function.

Among the four major species of carp in China, silver carp (*Hypophthalmichthys molitrix*) production represented 14.7% of the entire output of the cultured freshwater fish industry in 2020 [17]. Silver carp typically uses filter-feeding to capture phytoplankton, with no extra feeding. To date, silver carp spread or has been introduced to more than 88 countries worldwide, which has increased the breeding benefits and has also led to better control of algal blooms and enhanced water quality [18]. Silver carp is a pelagic fish that lives in the upper aquatic layer and is thus sensitive to changes in the DO content. In the high-temperature season, silver carp with high-density pond farming often experience increased pond turnover because of their low oxygen tolerance. Surprisingly, except for our previous study that only focused on the transcriptomic response to hypoxia in the silver carp brain [19], there has been a lack of studies focusing on the unfavorable effects of hypoxia–reoxygenation on the liver and brain of silver carp.

Proteomics can be used to describe molecular responses more directly than conventional transcriptomics, which assess only mRNA [20]. It is imperative to explore the damage to the liver and brain in *H. molitrix* using proteomics analysis to gain a deeper insight into the effects of hypoxia–reoxygenation. Therefore, in the present study, healthy silver carp were selected and exposed to acute hypoxia, followed by reoxygenation; the liver and brain functions were tested, after which TMT-based quantitative proteomics analysis was carried out. The results of the present study provide proteomic information concerning the molecular mechanism underlying the injury caused by hypoxia–reoxygenation to liver and brain tissues of fish. The data of this study could function as a guide to breed hypoxia-tolerant fish.

## 2. Materials and Methods

### 2.1. Experimental Animals

The seed multiplication farm of the Yangtze River Fisheries Research Institute in Jingzhou city, Hubei Province, China, provided the silver carp (2 years old, 65.42 ± 3.65 g in weight; 20.26 ± 1.21 cm in length). The fish were reared in a 400 L heated recirculating freshwater system and were permitted to acclimate for 7 d before the experiment. During acclimatization, the continuously aerated water was maintained at dissolved oxygen (DO) of 6.80 ± 0.50 mg/L; pH, 7.50 ± 0.20; Temperature, 25.00 ± 0.50 s °C; the ammonia-N and nitrite ranged from 0.005 to 0.020 mg/L. Throughout the experiments, the fish were subjected to a natural photoperiod (approximately 14 h light/10 h darkness).

### 2.2. Hypoxia–Reoxygenation Experiment and Sampling

After acclimation, 90 healthy fish were selected randomly and transferred into 3 tanks, with 30 fish in each tank. The pH and temperature were the same as those used during acclimatization. Nine fish (three fish from each tank), considered as the control group, were sampled before hypoxia exposure. The hypoxic conditions were set at the critical oxygen tension (Pcrit) of the experimental fish of 0.76 mg/L DO, determined according to a previously published method [21]. Hypoxic conditions were achieved by direct pumping of N_2_ into the water for approximately 20 min until the DO value reached 0.76 mg/L. The N_2_ supply was cut off, and the water was aerated slowly to maintain a DO of 0.76 ± 0.25 mg/L. Every 20 min, the DO value was monitored using a portable multiparameter meter (HACH, HQ40d, Loveland, CO, USA) and adjusted as required to maintain hypoxic conditions. Fish in the hypoxic group were subjected to hypoxic stress for 5 h, at which point the fish began to lose their equilibrium. Reoxygenation was then achieved by increasing the DO to 6.80 ± 0.50 mg/L, which was maintained for 5 h through continuous aeration (Figure S1A). Nine fish (three fish from each tank) from the hypoxia and reoxygenated groups were sampled at the time point of hypoxia and reoxygenation for 5 h, respectively.

The nine fish sampled from the three groups were euthanized using an overdose of MS-222 (90 mg/L, Sigma-Aldrich, St. Louis, MO, USA). After immediate tail amputation, blood samples were collected from each fish using a heparinized needle, and the serum was isolated from the blood and stored at 80 °C for further analysis. Simultaneously, the livers and brains from the three groups were collected (three replicates in each group, samples from three fish in each replicate were pooled), frozen immediately in liquid nitrogen, and stored at 80 °C until further processing.

### 2.3. Brain and Liver Function Tests

Serum was used to test liver functions. Commercial test kits from Nanjing Jiancheng Bioengineering Institute (Nanjing, China) were used to determine the serum alkaline phosphatase (ALP), glutamic-oxalacetic transaminase (AST), and glutamic-pyruvic transaminase (ALT) activities, and measure the serum total cholesterol (TC), globulin (GLB), albumin (ALB), and total protein (TP) concentrations, following the instructions of the kits. To evaluate brain functions, the concentrations of acetylcholine (ACh) and dopamine (DA) in the brain samples were measured using enzyme-linked immunosorbent assays (ELISAs; Nanjing Jiancheng Bioengineering Institute, Nanjing, China) according to the manufacturer’s instructions. Table S1 shows the product numbers of the kits.

### 2.4. Proteome Extraction and Digestion

Total proteins were extracted from the liver and brain samples using a previously described method [22]. A Bradford Protein Assay kit was used to determine the protein concentration of the final supernatants according to the manufacturer’s protocol.

### 2.5. TMT Labeling, Peptide Fractionation, and LC–MS/MS Analysis

TMT labeling of the obtained proteins was carried out according to a previously published method [23]. Reversed-phase chromatography fractionation of the TMT-labeled proteins was performed as described previously [23]. The obtained protein fractions were then analyzed using liquid chromatography with tandem mass spectrometry (LC–MS/MS), and the Novogene Co., Ltd. Company (Beijing, China) carry out the LC–MS/MS analysis using the method described by McBride et al. [24].

### 2.6. Bioinformatic Analyses

Proteome Discoverer software (Version PD1.4, Thermo Scientific, Waltham, MA, USA) was used to search the raw MS/MS data. Quantitative proteomics analysis was conducted according to a previously published protocol [25]. Principal coordinates analysis (PCoA) and Fuzzy clustering analysis were generated using OmicShare Tools (https://www.omicshare.com/tools/, accessed on 20 May 2021). The differentially abundant proteins (DAPs) between the test groups and the control group were identified with a fold change greater than 1.2 or less than 0.8 and *p* < 0.05 by a Mann–Whitney Test. The DAPs were subjected to functional analysis using the Cluster of Orthologous Groups of proteins (COG) database (https://www.ncbi.nlm.nih.gov/COG/, accessed on 23 May 2021) and Gene Ontology (GO) enrichment analysis (http://www.geneontology.org/, accessed on 27 May 2021). The Kyoto Encyclopedia of Genes and Genomes (KEGG) database (https://www.genome.jp/kegg/, accessed on 30 May 2021) was used to analyze enriched pathways associated with the DAPs. Protein functional domain analysis was performed using InterPro (http://www.ebi.ac.uk/interpro/, accessed on 30 May 2021). Subcellular localization of proteins was analyzed by Cell-mPLOC server (http://www.csbio.sjtu.edu.cn/bioinf/Cell-PLoc-2/, accessed on 5 June 2021). Prediction of protein–protein interaction (PPI) networks was conducted using the online analysis tool, STRING version 11 [26].

### 2.7. RNA Extraction and Quantitative Real-Time Reverse-Transcription PCR (qRT–PCR)

Total RNA was extracted from the backup materials for TMT, and its quality and quantity were determined using a previously published method [27]. The RNA was reverse transcribed to cDNA, which was then used as the template for the quantitative real-time PCR (qPCR) step of the qRT–PCR protocol [28], and the gene-specific primers (Table S2) used in this step were designed using the full-length transcriptome sequence of silver carp (unpublished). The samples were tested in triplicate, and the gene expression levels were normalized to that of *β-actin* [27]. The 2^−ΔΔCt^ method [29] was used to calculate the gene expression levels.

### 2.8. Statistical Analyses

Experimental measurements were repeated at least three times. The mean ± standard deviation (SD) was used to display the data. The data were analyzed statistically using one-way analysis of variance (ANOVA), followed by the Bonferroni multiple range test and Student’s *t*-test, using SPSS 19.0 software (IBM Corp., Armonk, NY, USA). *p* < 0.05 was considered significant.

## 3. Results

### 3.1. Acute Hypoxia and Reoxygenation Affects the Physiology of the Liver and Brain in H. molitrix

**Figure 1:**
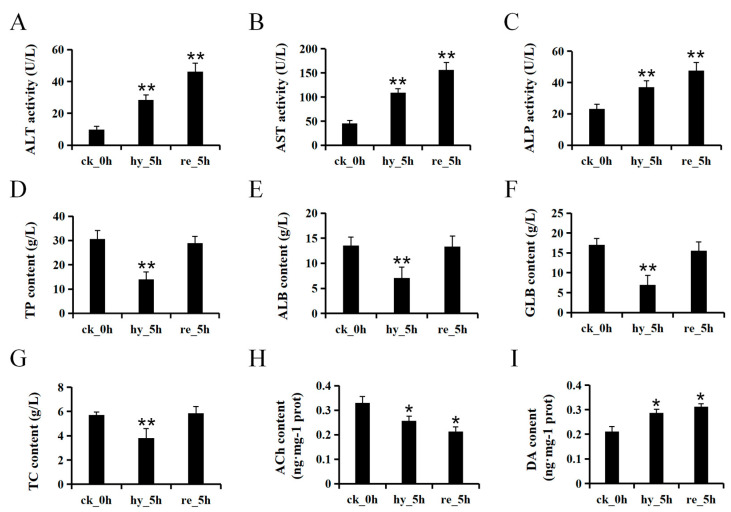
Alterations to biochemical indices related to the functions of the liver and brain in juvenile silver carp treated with hypoxia–reoxygenation. Values are expressed as the mean ± SD. The * *p* < 0.05 and ** *p* < 0.01 indicate significant differences compared with the control group. ck_0h: control group; hy_5h: hypoxia group; re_5h: reoxygenation group: (**A**–**C**) the serum ALT, AST, and ALP, activities; (**D**–**G**) the serum TP, ALB, GLB, and TC contents; (**H**,**I**) the brain Ach and DA contents. ALP, alkaline phosphatase; AST, glutamic-oxalacetic transaminase; ALT, glutamic-pyruvic transaminase; TC, total cholesterol; GLB, globulin; ALB, albumin; TP, total protein; Ach, acetylcholine; DA, dopamine.

The liver biochemical indices (ALP, AST, and ALT) were significantly different between the control and test groups. Compared with that in the control group, the serum ALT activity in the hypoxic group was 2.92-times higher, and that in the reoxygenated group was 4.74-times higher (Figure 1A). Similarly, the serum AST activities were 2.40-times (hypoxic group) and 3.43-times (reoxygenated group) higher than those in the control group (Figure 1B). Furthermore, a similar increasing trend was observed for the ALP activity, which was 1.60-times and 2.05-times higher in the hypoxic and reoxygenated groups, respectively, compared with that in the control group (Figure 1C). The levels of the key liver-synthesized proteins—TP, ALB, and GLB—were decreased in the hypoxia group compared with those in the control group (*p* < 0.01); however, during reoxygenation, they gradually returned to basal levels (Figure 1D–F). The TC level decreased significantly during hypoxia (*p* < 0.01) and then increased to that in the control group under reoxygenation (Figure 1G). The levels of two major neurotransmitters in the brain—Ach and DA—showed opposite tendencies during hypoxia and reoxygenation. Compared with that in the control group, hypoxia caused a 22.19% decrease in ACh and a 36.01% increase in DA, and these tendencies were further exacerbated by reoxygenation (Figure 1H,I).

### 3.2. Protein Identification, Quantification, and Annotation Analysis in the Liver and Brain of H. molitrix

Using TMT proteomics analysis (the flowchart of the method appears in Figure S1A), a total of 430,081 and 424,731 spectra were generated in the liver and brain, respectively. Among them, 114,829 and 124,776 spectra were matched to 62,273 and 72,499 peptides, and 6802 and 8358 proteins were identified, respectively, in the liver and brain. Ultimately, 6794 (99.88%) and 8353 (99.94%) proteins could be quantified in the liver and brain, respectively (Figure S1B, Table S3). The reliability of the proteomic data was demonstrated based on the distribution of peptide lengths, unique peptide numbers, and protein coverage (Figures S2–S4). Furthermore, the distribution of protein mass (Figure S5) in both the liver and brain revealed that 100% were above 10 kDa, of which more than 1000 and 1400 were above 100 kDa in the liver and brain, respectively. All of the quantified proteins in the liver and brain were annotated using GO, COG, KEGG, InterPro (IPR), and Cell-mPLOC (Figures S1C and S6–S10). The annotation data for all proteins obtained in this step were used as background information for subsequent data analysis (Tables S4 and S5).

### 3.3. Proteome Changes in the Liver and Brain in Response to the Acute Hypoxia and Reoxygenation Exposure

**Figure 2:**
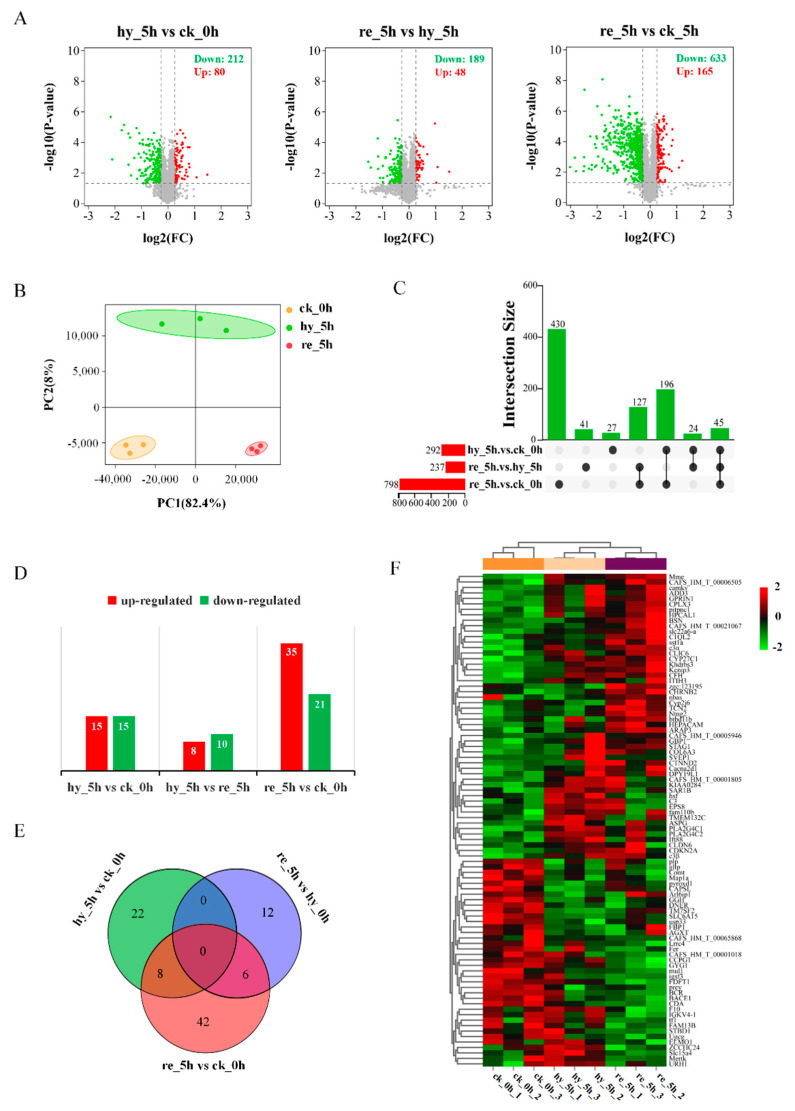
Differential protein expression analysis in the liver and brain among the control, hypoxia, and reoxygenation groups of juvenile silver carp: (**A**) volcano plots showing DAPs in pairwise comparisons in the liver; (**B**) principal coordinates analysis (PCoA) in the liver; (**C**) upset Venn plot for consistent DAPs in pairwise comparisons in the liver; (**D**) DAPs in pairwise comparisons in the brain; (**E**) Venn plot showing the common proteins shared in pairwise comparisons in the brain; (**F**) heat map displaying the relative expression of 90 DAPs in the brain. DAP, differentially abundant protein.

A 1.2-fold increase or decrease in the protein level and a *p*-value < 0.05 were used as criteria to identify DAPs. Figure 2 summarized the number and overlapping of DAPs in the liver and brain in response to acute hypoxia and reoxygenation.

After statistical analysis, 292 (80 increased and 212 decreased), and 30 (15 increased and 15 decreased) DAPs were identified in the liver and brain between the hypoxia group and the control group, respectively; 237 (48 increased and 189 decreased), and 18 (8 increased and 10 decreased) DAPs were identified in the liver and brain between the reoxygenation group and hypoxia group, respectively, whereas 789 (165 increased and 633 decreased), and 56 (35 increased and 21 decreased) DAPs were identified in the liver and brain between the reoxygenation group and the control group, respectively (Figure 2A,D; Tables S6 and S7). A Venn diagram was used to display the intersections among three datasets in the liver and brain (Figure 2C,E; Tables S8 and S9). Principal coordinates analysis (PCoA) showed a clear separation of proteins in the liver among the control group, hypoxia group, and reoxygenation group (Figure 2B). A heat map was used to display the relative expression of 90 total DAPs in the brain, which revealed substantial differences among the control, hypoxia, and reoxygenation groups (Figure 2F).

### 3.4. Bioinformatic Analyses of DAPs in the Liver

**Figure 3:**
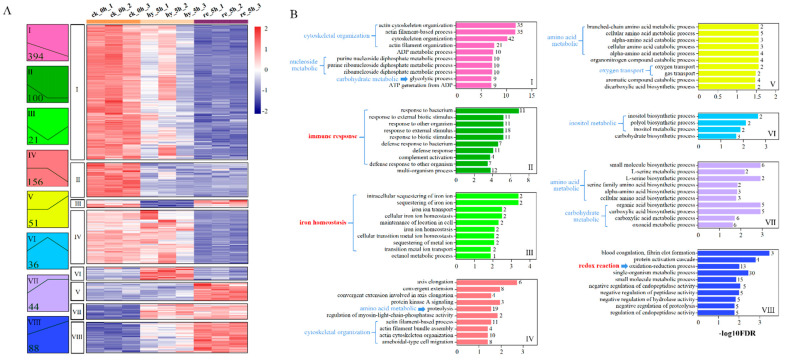
Co-expression modules of 890 DAPs in the liver of juvenile silver carp treated with hypoxia–reoxygenation: (**A**) abundance profiles of the eight statistically significant DAP clusters. Each model abundance profile is represented by a colored box. The boxes show the number of proteins categorized into each module. Heatmaps show the z-scores of the protein levels in the different samples; (**B**) GO biological process terms enriched for each cluster. Top 10 GO terms of each cluster are presented, and the number of DAPs for the terms are shown in the boxes. Ordinate displays log10 FDR (False discovery rate). DAP, differentially abundant protein; GO, Gene Ontology.

Through Fuzzy clustering analysis, a total of 890 DAPs between the hypoxia group and the control group, the reoxygenation group, and the hypoxia group, and the reoxygenation and the control group were divided into eight subclusters (Figure 3A), among which Cluster 1 (394, 44.27%) was the largest subcluster and contained DAPs that were downregulated.

DAPs in each cluster were classified into diverse functional categories using GO analysis, and the top 10 GO terms of each cluster are presented in Figure 3B (only four GO terms were statistically significant in Cluster 6, *p* < 0.05). When analyzing the GO terms, we found that 42 DAPs in Cluster 1 and 11 DAPs in Cluster 4 were enriched in a cytoskeletal organization (Table S10), 19 DAPs in Cluster 2 were enriched for the immune response, 2 DAPs in Cluster 3 were enriched for iron homeostasis, and 13 DAPs in Cluster 8 were enriched for redox reactions (Table 1). Otherwise, the metabolic process was significantly enriched in most clusters, including nucleoside metabolic, amino acid metabolic, carbohydrate metabolic, and inositol metabolic functions.

**Figure 4:**
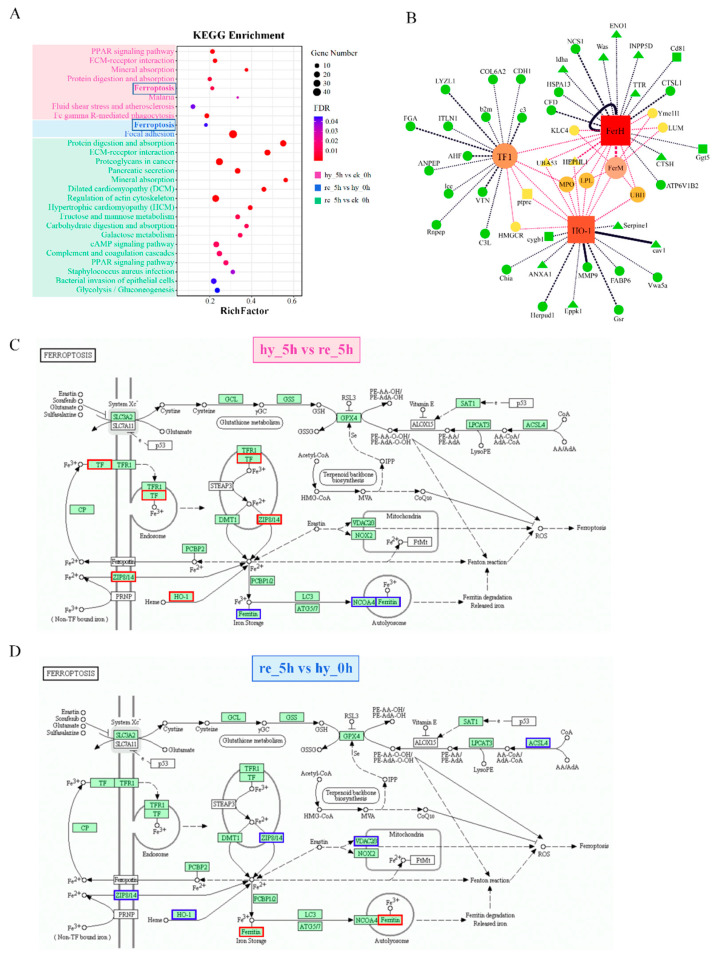
KEGG pathway analysis showing ferroptosis in liver under hypoxia-reoxygenation: (**A**) enriched KEGG pathways of pairwise comparisons in the liver; (**B**) protein–protein interaction (PPI) network shows the interaction between the total DAPs and the DAPs involved in ferroptosis in the liver. Circles represent DAPs between the hypoxia group and the control group, triangles represent DAPs between the reoxygenation group and the hypoxia group, and squares represent the DAPs common to both comparisons. The thicker the line, the higher the correlation coefficient. The larger the dot, the higher the number of proteins in the interaction; a more centrally placed network has a more important or critical function; (**C**) six DAPs between the hypoxia group and the control group were enriched in the ferroptosis pathway; (**D**) five DAPs between the reoxygenation group and the hypoxia group were enriched in the ferroptosis pathway. Blue boxes represent downregulated proteins, and red boxes represent upregulated proteins. DAP, differentially abundant protein; KEGG, Kyoto Encyclopedia of Genes and Genomes.

KEGG pathway analysis was performed for the DAPs between (1) the hypoxia group and the control group, (2) the reoxygenation group and the hypoxia group, and (3) the reoxygenation and the control group. Figure 4A shows all the significantly enriched KEGG pathways for the above pairwise comparisons. Among them, three pathways were involved in the cellular community, motility, proliferation, and survival, including ECM–receptor interaction, focal adhesion, and regulation of actin cytoskeleton; whereas two pathways were involved in iron homeostasis, including mineral absorption and ferroptosis, and steroid hormone biosynthesis. Complement and coagulation cascades, staphylococcus aureus infection, and bacterial invasion of epithelial cells, which are involved in the immune response, were also enriched. In addition, the metabolism-related processes, glycolysis/gluconeogenesis, galactose metabolism, and fructose and mannose metabolism were enriched.

Notably, six DAPs between hypoxia group and control group—namely, serotransferrin-1 (TF1), heme oxygenase-1 (Hmox-1/HO-1), nuclear receptor coactivator 4 (NCOA4), zinc transporter ZIP8 (SLC39A8/ZIP8), ferritin, and middle/heavy subunit (FerM and FerH)—were enriched in ferroptosis. Furthermore, ferroptosis was enriched between the reoxygenation group and hypoxia group, with five DAPs—namely, HO-1, FerH, ZIP8, voltage-dependent anion-selective channel protein 2 (VDAC2), and acyl-CoA synthetase long-chain family member 4 (ACSL1). Interestingly, HO-1 and ZIP8 were upregulated during hypoxia, whereas they were downregulated during reoxygenation, and FerH was downregulated during hypoxia but upregulated during reoxygenation (Figure 4C,D). A PPI network was constructed to assess the protein interactions of DAPs, which were interacted with the above-mentioned ferroptosis-related proteins (Figure 4B). The result showed that 32 proteins are involved in 68 interactions during hypoxia and reoxygenation, indicating the complex response processes in ferroptosis.

### 3.5. Bioinformatic Analyses of DAPs in the Brain

**Figure 5:**
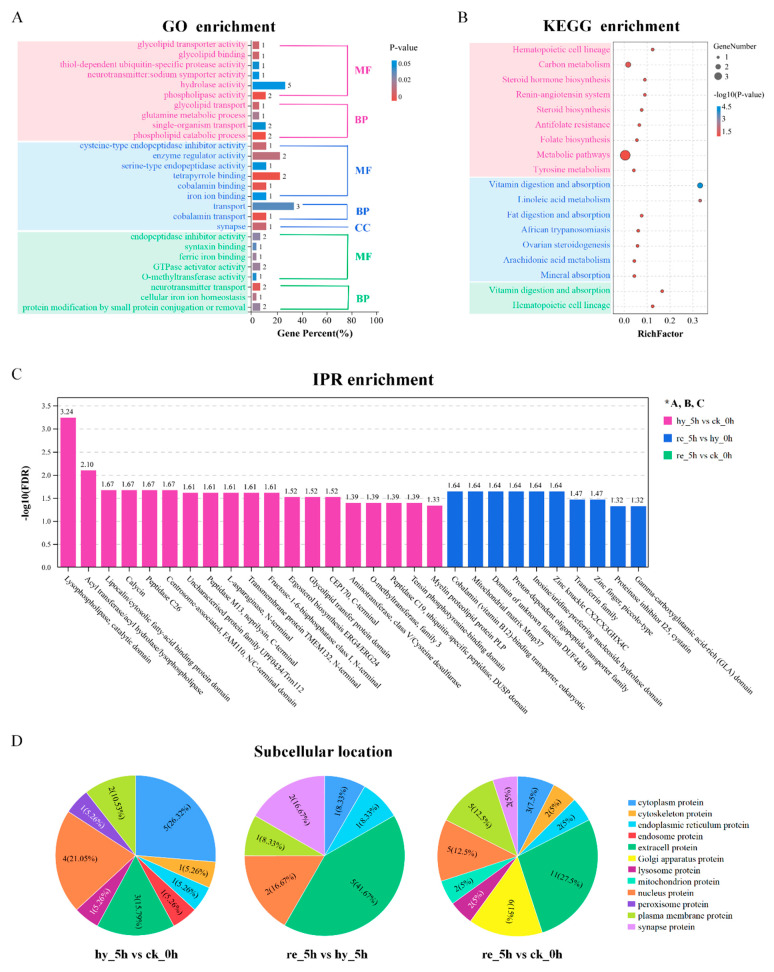
Bioinformatic analyses of DAPs in the brain: (**A**–**C**) GO, KEGG pathway, and IPR enrichment analyses of DAPs obtained by pairwise comparisons in the brain; (**D**) subcellular location analysis of DAPs obtained by pairwise comparisons in the brain. DAP, differentially abundant protein; GO, Gene Ontology; KEGG, Kyoto Encyclopedia of Genes and Genomes; IPR, InterPro.

To functionally classify the DAPs in response to hypoxia and reoxygenation treatment in the brain, GO analysis was performed with the functional categories of biological processes (BP), cellular components (CC), and molecular functions (MF). All annotated GO terms (*p* < 0.05) are shown in Figure 5A. When comparing the hypoxia group and the control group, phospholipid catabolic process and phospholipase activity were the most represented terms, followed by glycolipid transporter and glycolipid binding. Meanwhile, glutamine metabolic process, neurotransmitter: sodium symporter activity, and hydrolase activity were also enriched. When comparing the reoxygenation group and the hypoxia group, DAPs were most significantly enriched in tetrapyrrole binding, cobalamin binding, and cobalamin transport, followed by cysteine-type endopeptidase inhibitor activity and synapse. Other enriched GO terms between the reoxygenation and hypoxia groups covered synapse, transport, serine-type endopeptidase activity, and iron ion binding. When comparing the reoxygenation group and the control group, the annotated GO terms of the DAPs included neurotransmitter transport, cellular iron ion homeostasis, ferric iron binding, endopeptidase inhibitor activity, syntaxin binding, and O-methyltransferase activity.

The brain-derived DAPs were subjected to KEGG enrichment analysis, and nine, seven, and two pathways were significantly enriched in the three comparisons, respectively (Figure 5B). Among them, hematopoietic cell lineage, renin–angiotensin system, steroid hormone biosynthesis, tyrosine metabolism, steroid biosynthesis, antifolate resistance, and folate biosynthesis were enriched under hypoxia stress, while vitamin digestion and absorption, linoleic acid metabolism, ovarian steroidogenesis, arachidonic acid metabolism, and mineral absorption were enriched under reoxygenation conditions. IPR enrichment analysis was applied to the DAPs, and the results showed that 19 and 10 significantly enriched functional domains were identified between the hypoxia and control groups, and the reoxygenation and hypoxia groups, respectively (Figure 5C). Subcellular location analysis was performed for the DAPs (Figure 5D). Cytoplasmic proteins (five DAPs, 26.32%) and nuclear proteins (four DAPs, 21.05%) were most significantly enriched between the hypoxia and control groups, whereas extracellular proteins, synapse proteins, and Golgi apparatus proteins were increased during reoxygenation.

**Figure 6:**
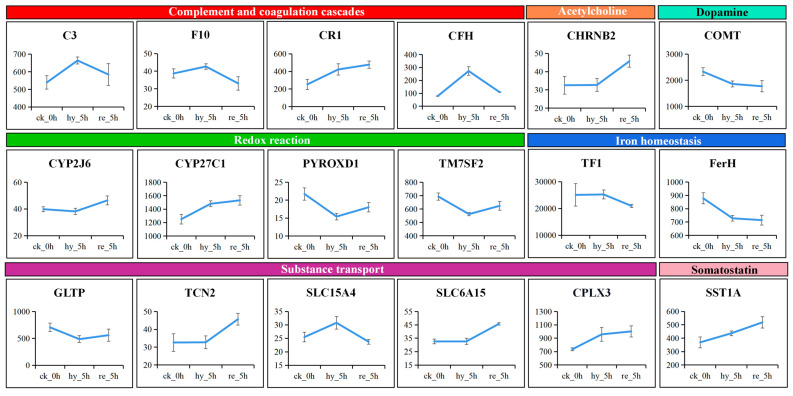
The changes in abundance of 18 DAPs in brain. The ordinate represents the TMT-based quantification values. They were classified according to their roles in immune response, iron homeostasis, hormone and neurotransmitter synthesis, Redox reaction, and substance transport. DAP, differentially abundant protein.

Based on the results of functional enrichment analysis, we selected 18 DAPs in the brain and classified them into several categories according to their roles in the immune response, iron homeostasis, substance transport, hormone, and neurotransmitter synthesis. The changes in abundance of the 18 DAPs are presented in Figure 6. Studying these changes might help us to understand how the brain responds to hypoxia and reoxygenation.

### 3.6. Validation of the DAPs Identified from the Proteomics Analysis

To validate the quality of the proteomic data and the reliability of the DAPs, qRT–PCR analysis was conducted. A total of 18 identified DAPs were selected at random in the liver and brain, respectively. The expression trends of these genes detected by qRT–PCR were in good agreement with the fold changes obtained from proteomics analysis (Figure S11). These results indicated that, for these 36 proteins, the mRNA changes and protein changes are tightly coupled, demonstrating the reliability of the proteomic data to a certain extent.

## 4. Discussion

The results of the present study showed that hypoxia–reoxygenation induced damage to the brain and liver. The recurring hypoxia–normoxia cycle induces different damages from that induced by once-occurring hypoxia followed by reoxygenation, and we focused on just the once-occurring hypoxia–reoxygenation in this study. Immune response, iron homeostasis, and redox reactions were identified as being involved in the once hypoxia and reoxygenation responses. Interestingly, ferroptosis was found to be activated under hypoxia and inhibited under reoxygenation in the fish liver. Ferroptosis is a type of iron-dependent regulated cell death mediated by the lethal accumulation of lipid peroxides [30], and it is always caused by environmental stressor stimuli [31]. The mechanisms of ferroptosis have become a hot topic in recent years in mammals [32]; however, they have received scant attention in teleost fish.

### 4.1. Hypoxia–Reoxygenation Impairs the Function of Liver and Brain

ALT, AST, and ASP are mainly found in liver cells, and their release into serum from the liver indicated increased permeability and damage in the liver, such as hepatocyte necrosis and dysfunction in the cell membrane. In Tilapia (*Oreochromis niloticus*), hypoxia increased the serum ALT and AST activities significantly and reoxygenation increased the ALT activity further [33]. Interestingly, the serum AST and ALT activities increased markedly during reoxygenation, suggesting worsening liver cell damage under reoxygenation (Figure 1A,B). This could be the result of hypoxia–reoxygenation injury or might represent a lag in the effect of hypoxia. In the liver of *Lateolabrax maculatus*, cell death was observed to be induced soon after reoxygenation [10]. The dying cells underwent autolytic disintegration, which increased cytoplasmic membrane permeability, causing the release of liver transaminase into the serum.

Globulin (GLB) and albumin (ALB) are the major components of the serum total proteins (TP). Cholesterol is an essential structural component of the plasma membrane, also serves as the substrate for the synthesis of steroid hormones, vitamin D, and bile acids. Synthesis and degradation of cholesterol mainly occur in the liver. In *H. molitrix*, a hypoxia-sensitive fish, the liver’s protein and cholesterol synthesis function was inhibited during hypoxia but was restored during reoxygenation (Figure 1D–F). This observation was similar to a report on rainbow trout (*Oncorhynchus mykiss*), in which hypoxic conditions changed the expression of protein metabolic markers, implying a shutdown of protein synthesis in the liver [34]. Similarly, in the hypoxia-tolerant fish crucian carp [35] and Amazonian cichlid (*Astronotus ocellatus*) [36], liver protein synthesis decreased during hypoxia but recovered during reoxygenation. To survive under hypoxic conditions, crucian carp underwent metabolic depression to reduce its ATP consumption, thus avoiding the rapid depletion of glycogen and a marked reduction in cellular ATP levels [35]. In fish, 23–53% of total oxygen is consumed by protein synthesis; a reduction in the synthesis of protein might contribute markedly to the maintenance of energy homeostasis [37].

In contrast to the liver, brain protein synthesis was maintained, probably because survival relies on protein synthesis, and under ischemic conditions, neural necrosis occurs rapidly (within 4 to 5 min) from onset [35]. However, key detrimental events in the oxygen-deficient brain, such as alterations of several neurotransmitters, appear to be very similar in fish and mammals [11]. Neurotransmitters are responsible for synaptic signaling transmission, and the main neurotransmitter systems include the cholinergic system and the dopaminergic system [38]. Acetylcholine (Ach) is widely distributed in the nervous system and has been implicated to play a critical role in cerebral cortical activity and sleep–wake cycle, as well as in modulating cognitive performances and learning/memory processes. Dopaminergic neurons, which are located in the ventral midbrain, are essential for the control of diverse cognitive and motor behaviors and associated with multiple psychiatric and neurodegenerative disorders. In the present study, we observed that ACh levels showed a decreasing trend during hypoxia and reoxygenation, which was in accordance with previous reports in mammals [39]. Inhibition of the cholinergic system is believed to result in contractions of hypoactive muscles and decreased locomotion [38]. Furthermore, the level of neuronal acetylcholine receptor subunit beta-2 (CHRNB2) showed an increasing trend from hypoxia to reoxygenation (Figure 6), indicating a compensatory effect in response to the reduced synthesis of acetylcholine. This is consistent with previous findings showing that when the content of ACh decreased, nicotinic acetylcholine receptor α-7 (α-7nAChR), which is encoded by *chrnα7*, accumulated [40]. The brain DA concentration showed an increasing tendency after treatment with hypoxia and reoxygenation, which compared favorably with the results of a previous study [41]. This result corresponds with the proteome data showing that the level of catechol O-methyltransferase (COMT) continued to decline (Figure 6). COMT plays an active role in the metabolism of DA in the prefrontal cortex, and a marked increase in prefrontal dopamine pools was observed in *Comt*-knockout mice [42]. DA plays an inhibitory role in the neuroendocrine regulation of the last steps of gametogenesis in adult teleosts [43], indicating that hypoxia–reoxygenation conditions might damage the reproductive system and reduce the sperm and egg quality of *H. molitrix*.

### 4.2. Effects of Hypoxia–Reoxygenation on the Immune Response, Iron Homeostasis, and Redox Reactions in the Liver and Brain

#### 4.2.1. Immune Response

Hypoxia has been shown to modify the innate and adaptive immune responses in fish, prejudicing the immune system and leading to lower resistance to pathogen infections [44]. In the present study, we found that reoxygenation could not improve the compromised immunity in the liver. Overall, 19 immunity-related proteins belonging to Cluster 2 were significantly downregulated (Figure 3). Among them, complement is an important component of the innate immune system, and complement C3 (C3) and complement factor B (CFB) are central members in the complement system [45]. All complement activation cascades converge on the point at which C3 is cleaved into anaphylatoxin C3a and opsonin C3b [46]. CFB is an important soluble component, which binds to C3b and is then cleaved into fragments Ba and Bb, ultimately forming the convertase, C3bBb. C3bBb is the key enzyme that cleaves more C3 to C3b, generating an amplification loop to activate the complement pathway [47]. Myeloperoxidase (MPO), a heme protein, is a major neutrophil component that contributes to the induction and maintenance of an alkaline environment, which is optimal to combat microbes [48]. Matrix metalloproteinase-9 (MMP9) belongs to a family of zinc-dependent endopeptidases that cleave structural extracellular matrix molecules. Reports have noted that MMP9 is associated with vital inflammatory processes, such as leucocyte migration and tissue remodeling and regeneration [49]. High-mobility group box 3 (HMGB3) is a universal sentinel in the activation of innate antiviral immune responses in mammalian cells. A study provided evidence that HMGB3 participates in broad antiviral and antibacterial immune responses in teleosts [50]. In addition, ephrin-B1 (EFNB1) plays an important role in T cell development and function, and caspase-1 (CASP1), as a unique cysteine protease, plays a central role in innate immunity. The downregulation of these important immune-related proteins implied reduced immune capacity in *H. molitrix* under hypoxia–reoxygenation.

For years, scholars believed that the brain was an immune-privileged organ [51]. No significant enrichment was found for immune-correlated process GO terms and KEGG pathways in the brain in our study. However, members of the complement cascade, including C3, coagulation factor X (F10), complement component (3b/4b) receptor 1 (CR1), and complement factor H (CFH) were upregulated under hypoxia. Except for CR1, the expression of all these proteins returned to their baseline levels after reoxygenation. These results suggested that the hypoxia-induced brain injury might have induced an inflammatory response, and the complement system participated in the regulation of this inflammation [52].

#### 4.2.2. Iron Homeostasis

Iron is an integral component of the heme porphyrin ring and thus is essential for the oxygen supply to cells and tissues. Iron is almost completely insoluble and has the potential for physiological toxicity; therefore, organisms have evolved specialized molecular mechanisms to acquire, transport, and store iron in a nontoxic and soluble form. Typically, cells acquire iron from a well-characterized plasma glycoprotein, transferrin (TF), and most of the transferrin-bound iron is used to synthesize hemoglobin [53]. Excess iron in the intracellular labile pool that is not required to synthesize heme and nonheme iron-containing proteins is stored within the structure of the iron-storage protein, ferritin (Fer), preventing the generation of free radicals via the Fenton reaction. In the ferritin cage, Fe(II) is oxidized to Fe(III), which is unavailable for use or the generation of reactive oxygen species (ROS). When intracellular free iron levels fall, ferritin is broken down by the lysosome, where Fe(III) is converted into Fe(II), for iron release and cellular iron utilization [54]. This remarkable regulatory mechanism, which involves important iron homeostasis proteins, such as TF, the transferrin receptor (TFR), heme oxygenase-1 (HO-1), and Fer, prevents the catalytically active intracellular iron pool from expanding while maintaining metabolically sufficient iron levels.

It has been reported that under hypoxia, hypoxia-inducible factors (HIFs) activate TF, TFR [55], and HO-1 [56] to increase the availability of iron for erythropoiesis to enhance the uptake and delivery of oxygen to hypoxic cells. In our study, a similar situation was observed in the liver, TF1, and HO-1 levels were upregulated under hypoxia and then downregulated after reoxygenation (Figure 4C,D). Fer, including FerH and FerM, levels were downregulated under hypoxic conditions to release the iron into the cytoplasm of liver cells and were then upregulated when the oxygen supply was restored (Table 1; Figure 3B and Figure 4C,D). Fer might be downregulated via autophagic degradation, a process that is regulated by the autophagy receptor, NCOA4 [57]. NCOA4, which binds both LC3/GABARAPs and ferritin, demonstrated identical abundance trends to Fer under hypoxia (Figure 4C). In addition, *SLC39A8/ZIP8*, an evolutionarily highly conserved gene that encodes the ZIP8 metal cation transporter, was upregulated under hypoxia, presumably to move ferrous iron into the liver cells, and downregulated thereafter (Figure 4C,D). This series of changes under hypoxia pointed toward the elevation of intracellular iron concentrations and enhanced heme biosynthesis, which also triggered intracellular damage at the same time. Ferroptosis is a newly identified process of programmed necrosis whose initiation requires iron-dependent accumulation of cellular ROS [58]. Ferroptosis results from the loss of cellular redox homeostasis, and lipid ROS, rather than cytosolic ROS, appears to have a more important function in ferroptosis. The ferroptosis pathway was enriched under both hypoxic and reoxygenation conditions (Figure 4A). Under hypoxia, ferroptosis was activated for the induction of cellular iron accumulation, and under reoxygenation, ferroptosis was inhibited. As shown in Figure 4D, the downregulation of HO-1 and ZIP8, as well as the upregulation of Fer, indicated a reduction in cytosolic free iron. Moreover, erastin was reported to induce ferroptosis by binding directly to VDAC2/3, which changes the outer mitochondrial membrane’s permeability, thereby reducing the rate of NADH oxidation (NOX) [59]. In addition, *VDAC2* or *VDAC3* knockdown resulted in erastin resistance [60]. ACSL4, which is involved in lipid peroxidation, is an essential component for ferroptosis execution, and *Gpx4-Acsl4* double-knockout cells showed marked resistance to ferroptosis [61]. In our study, VDAC2 and ACSL4 levels were both downregulated under reoxygenation (Figure 4D), which further demonstrated that ferroptosis was inhibited when the oxygen supply was restored.

Unlike the liver, ferroptosis was not found in the brain during hypoxia–reoxygenation. TF1 was not upregulated under hypoxia, whereas FerH was downregulated to promote iron release and Heme synthesis (Figure 6). During reoxygenation, FerH was no longer significantly downregulated, while TF1 level was significantly decreased (Figure 6), and these changes were associated with an adequate oxygen supply. The above results demonstrated that acute hypoxia disrupted iron homeostasis and intracellular ROS to induce ferroptosis in the liver but merely raised the intracellular ferric ion concentration felicitously to promote Heme synthesis in the brain. We speculated that the preferential perfusion of the brain under hypoxia might have contributed to this phenomenon.

#### 4.2.3. Redox Reactions

The reactions that ROS and reactive nitrogen species (RNS) engage in are termed reduction–oxidation (redox) reactions. If not eliminated by antioxidants, ROS and RNS, as highly reactive compounds, will react with and potentially alter the structure and function of several cellular components, such as cell membranes, cellular proteins, carbohydrates, DNA, and RNA, and this condition is termed oxidative stress [62]. Reduced glutathione (GSH) is a tripeptide consisting of glutamate, cysteine, and glycine, and is present in nearly all cells at high concentrations. The ratio of oxidized and reduced glutathione (GSH/GSSG) is a good biomarker of oxidative stress intensity. Usually, a relatively high ratio is maintained intracellularly, but under oxidative conditions, it can be lowered because GSH is oxidized into GSSG by ROS. Reports have noted that acute hypoxia and reoxygenation can lead to oxidative stress in fish liver and brain [63]. In our present study, hypoxia and reoxygenation significantly reduced GSH and T-GSH/GSSH in the brain and liver (Table S11), indicating that hypoxia–reoxygenation induced oxidative stress. Moreover, 13 oxidation–reduction-related proteins were upregulated in the liver during hypoxia–reoxygenation (Table 1, Figure 3B). Glutathione peroxidase 4 (GPX4) can prevent ferroptosis through clearance of lipid peroxides [64], and the upregulation of GPX4 level indicated a general resistance to ferroptosis in the liver. The level of the stress-responsive isoenzyme HO-1 was upregulated under hypoxia. Besides assisting in ionic release, HO-1 provides protection against programmed cell death, relying on its ability to catabolize free heme and prevent it from sensitizing cells [65]. The large family of versatile enzymes responsible for metabolizing most drugs and many toxicologically important chemicals is given the generic name cytochrome P450 (CYP450). In addition, CYP450 enzymes have vital functions in various physiological processes, such as ion homeostasis maintenance, fatty acid metabolism, and steroid and cholesterol biosynthesis [66]. In the present study, the levels of six members of the CYP450 family were upregulated during hypoxia–reoxygenation (Table 1)—namely, CYP1A1, CYP3A27, CYP4V2, CYP4V3, CYP8B1, and CYP39A1. When exposed to a hostile environment, CYP1A1 expression was reported to be elevated under regulation by the aryl hydrocarbon receptor (AhR), resulting in ROS induction [67]. In a previous study, using CYP4V2 mutant pluripotent stem (RPE) cells as disease models, researchers demonstrated that RPE cells with CYP4V2 mutations accumulated an excessive amount of polyunsaturated fatty acid (PUFA), which in turn promoted ROS production [68]. Tanaka et al. found that compared with wild-type mice, the basal expression of *Cyp8b1* was lower in *Nrf2* (encoding NF-E2-related factor 2)-null mice, indicating that CYP8B1 is involved in the inhibition of lipid accumulation and oxidative stress [69]. Taken together, the upregulated CYP450 family members might play important roles in lipid metabolism and oxidative stress induced by hypoxia–reoxygenation in the liver.

The levels of CYP450 family members CYP2J6 and CYP27C1 were also upregulated during hypoxia–reoxygenation in the brain and were enriched in the GO terms of iron ion binding and ferric iron binding, respectively, suggesting that they participate in the regulation of iron homeostasis. In addition, the levels of pyridine nucleotide-disulfide oxidoreductase domain 1 (PYROXD1) and transmembrane 7 superfamily member 2 (TM7SF2) were downregulated under hypoxia but recovered to some degree under reoxygenation. PYROXD1 is a ubiquitously expressed protein containing an oxidoreductase domain, and functional investigations in yeast and mammalian cell models provided evidence of a reductase activity that can antagonize the effects of oxidative stress [70]. *Tm7sf2* encodes an endoplasmic reticulum enzyme, and its subcellular localization suggests a primary role in cholesterol biosynthesis. Affymetrix microarray analysis revealed that several genes involved in xenobiotic metabolism, including those encoding CYP450 enzymes and glutathione-S-transferase (GST), are upregulated in *Tm7sf2*^(−/−)^ mice [71]. Thus, the changes in PYROXD1 and TM7SF2 levels under hypoxia hinted that they might be involved in the induction of, or resistance to, oxidative stress.

### 4.3. Other Effects of Hypoxia–Reoxygenation on the Liver

All life activities require energy, the production of which uses oxygen. Therefore, when an organism experiences hypoxia, changes in energy metabolism occur, especially in the liver, the major organ for energy metabolism [72]. In organisms, the three principal sources of energy are protein, lipids, and carbohydrates. In the present study, GO terms and KEGG pathways related to carbohydrate metabolism and amino acid metabolism were enriched in the liver (Figure 3B and Figure 4A); however, no GO terms or KEGG pathways related to lipid metabolism were significantly enriched. The oxidative breakdown of fatty acids consumes a large amount of oxygen to produce energy; therefore, lipid metabolism is modified during hypoxia in a HIF-dependent manner. Previous studies have shown that energy production using lipids as a substrate decreases markedly in hypoxia, and severe hypoxia could induce lipid accumulation [72], which fits the results of the present study.

Currently, increasing evidence has demonstrated that hypoxia induces cytoskeleton disruption [8], and in our study, we found that reoxygenation could exacerbate this process in the liver. As shown in Figure 3B and Table S10, 42 DAPs involved in the cytoskeletal organization were downregulated throughout hypoxia–reoxygenation. Moreover, a further 11 DAPs involved in cytoskeletal organization were downregulated from the onset of reoxygenation. These DAPs were enriched in Focal adhesion and ECM–receptor interaction KEGG pathways (Figure S12), which regulate cell motility, cell proliferation, and cell survival.

### 4.4. Other Effects of Hypoxia–Reoxygenation on the Brain

Hypothalamus-produced somatostatin (SST) is transported to the anterior pituitary gland, where it inhibits constant growth hormone (GH) secretion and is responsible for its pulsatile release [73]. In our study, somatostatin-1A (SST1A) levels were upregulated under hypoxia and continued to increase following the restoration of oxygen supply (Figure 6). This finding supported the speculation that hypoxia in fish can lead to adverse effects on growth. Five DAPs were enriched in GO terms associated with transport (Figure 5A), including glycolipid transfer protein (GLTP, GO:0017089: glycolipid transport), transcobalamin-2 (TCN2, GO:0015889: cobalamin transport), solute carrier family 15 member 4 (SLC15A4, GO:0006857: oligopeptide transport), solute carrier family 6 member 15 (SLC6A15, GO:0005328: neurotransmitter: sodium symporter activity), and complexin-3 (CPLX3, GO:0006836: neurotransmitter transport). GLTP is a small (23–24 kDa) basic protein that dramatically accelerates the specific intermembrane transfer of neutral glycosphingolipids and ganglioside [74]. Cobalamin is an essential cofactor for many biochemical pathways, and TCN2 is required to internalize cobalamin into the cells through membrane receptor-mediated endocytosis. Deficiency of TCN2 results in an elevation in methylmalonic acid and homocysteine, leading to neurological dysfunction [75]. SLC15A4 is responsible for translocating free histidine and certain di/tripeptides in the brain, resulting in effects on histidine/histamine regulation and neuropeptide homeostasis [76]. SLC15A4 was suggested to participate in the removal of degraded neuropeptides, e.g., neuromodulators from the synaptic cleft, and the might control oligopeptide uptake to regulate central nervous system (CNS) cellular metabolism [77]. The SLC6A15 transporter is almost exclusively expressed in the CNS and has been functionally characterized as a Na^+^-coupled amino acid transporter [78]. Previous research provided supportive roles for SLC6A15 in modulating anxiety and depressive-like behavior [79]. CPLX3 regulates synaptic vesicle fusion speed and Ca^2+^ sensitivity and is necessary for the maintenance of synaptic structures in the retina; indeed, its loss caused vision deficits [80]. Thus, these transporters were related to the nervous system, including neuronal development and differentiation, neurotransmitter release, and synaptogenesis. Consequently, changes in expression of these transporters (Figure 6) in silver carp might disrupted the organization of the brain, with a consequently higher risk of neurodegenerative disorders.

## 5. Conclusions

In this study, hepatic biochemical activity indices in serum significantly increased under hypoxia and continued to increase following the restoration of oxygen supply, indicating that normal liver function of silver carp might be impaired by hypoxia and become worse after reoxygenation. The major neurotransmitters contents in the brain were markedly altered after acute hypoxia exposure and reoxygenation for a short time, showing that hypoxia–reoxygenation conditions might damage the nervous system of silver carp. In addition, TMT-based quantitative proteomics analysis was conducted to assess the molecular effects of hypoxia–reoxygenation on the brain and liver of silver carp, which identified a total of 8353 and 6794 proteins in the brain and liver, respectively. Proteomics analysis of the liver and brain highlighted that hypoxia–reoxygenation had adverse effects on the growth, locomotion, immunity, and reproduction of silver carp, and could damage the functions of the liver and brain, which might be caused by ferroptosis, oxidative stress, and cytoskeleton destruction in the liver, and abnormal expression of susceptibility genes for neurodegenerative disorders in the brain. The findings of the current study partially explain the mechanism of hypoxic and reoxygenated injury in the brain and liver of fish that are sensitive to hypoxia. Moreover, our results could act as a guide to breed hypoxia-tolerant fish.

## Figures and Tables

**Table 1:** DEPs involved in immune response, iron homeostasis, and redox reaction in the liver under hypoxia–reoxygenation.

	ID	Protein Name	Annotation
immune response (19)	CAFS_HM_T_00069923	C3	Complement C3 (Fragment)
	CAFS_HM_T_00069924	C3	Complement C3 (Fragment)
	CAFS_HM_T_00051668	CFB	Complement factor B
	CAFS_HM_T_00051663	CFB	Complement factor B
	CAFS_HM_T_00004034	MPO	Myeloperoxidase
	CAFS_HM_T_00004821	mmp13	Collagenase 3 (Fragment)
	CAFS_HM_T_00038844	MMP9	Matrix metalloproteinase-9
	CAFS_HM_T_00009179	rnasel3	Ribonuclease-like 3
	CAFS_HM_T_00024834	HMGB3	High-mobility group protein B3
	CAFS_HM_T_00051562	RHOG	Rho-related GTP-binding protein RhoG
	CAFS_HM_T_00059585	LYZL1	Lysozyme-like protein 1
	CAFS_HM_T_00068155	EFNB1	Ephrin-B1
	CAFS_HM_T_00070745	CASP1	Caspase-1
	CAFS_HM_T_00085126	CASP1	Caspase-1
	CAFS_HM_T_00075145	GLUL	Glutamine synthetase
	CAFS_HM_T_00078382	dapl1-b	Death-associated protein-like 1-B
	CAFS_HM_T_00086469	alcama	CD166 antigen homolog A
	CAFS_HM_T_00063121	--	Histone H1
	CAFS_HM_T_00071322	--	Nattectin
iron homeostasis (2)	CAFS_HM_T_00047854	FerH	Ferritin, heavy subunit
	CAFS_HM_T_00015421	FerM	Ferritin, middle subunit
redox reaction (13)	CAFS_HM_T_00005616	Gpx4	Phospholipid hydroperoxide glutathione peroxidase, mitochondrial
	CAFS_HM_T_00018176	HO-1	Heme oxygenase-1
	CAFS_HM_T_00009561	CYP1A1	Cytochrome P450 1A1
	CAFS_HM_T_00072318	CYP3A27	Cytochrome P450 3A27
	CAFS_HM_T_00037413	CYP4V2	Cytochrome P450 4V2
	CAFS_HM_T_00069566	CYP4V3	Cytochrome P450 4V3
	CAFS_HM_T_00076880	CYP8B1	Sterol 12α-hydroxylase
	CAFS_HM_T_00031504	CYP39A1	Oxysterol 7α-hydroxylase
	CAFS_HM_T_00048267	ACOX3	Peroxisomal acyl-coenzyme A oxidase 3
	CAFS_HM_T_00021903	DEGS1	Sphingolipid delta(4)-desaturase DES1
	CAFS_HM_T_00044883	HSD11B2	Corticosteroid 11-beta-dehydrogenase isozyme 2
	CAFS_HM_T_00037168	Tdo2a	Tryptophan 2,3-dioxygenase A
	CAFS_HM_T_00086195	Tmem195	Alkylglycerol monooxygenase

## Data Availability

Data are contained within the article and Supplementary Materials.

## Supplementary Materials

The following supporting information can be downloaded at: www.mdpi.com/article/10.3390/antiox11030589/s1, Figure S1: Analysis of the proteome in the liver and brain: (A) a schematic diagram of the experimental procedure. Pcrit, The critical oxygen tension of the experimental fish; (B) the total spectra, peptides, and proteins identified in the liver and brain, respectively; (C) Venn diagram of the statistics of the functional annotation in the liver and brain, Figure S2: Peptide length distribution map of the identified peptides in liver (A) and brain (B). The abscissa indicates the amino acid residue number and the ordinate is the peptide number in the length of each peptide. The peptides length is mainly distributed in 7–25, Figure S3: Unique peptide number distribution map of the identified peptides in liver (A) and brain (B). The abscissa indicates the number of Unique peptides and the ordinate is the ratio of protein containing Unique peptides to total protein, Figure S4: Protein coverage distribution map in liver (A) and brain (B). The abscissa indicates the protein coverage interval (the length of protein covered by detected peptide/full-length the protein) and the ordinate is the protein number in each interval, Figure S5: Protein mass distribution map in liver (A) and brain (B). The abscissa indicates the mass of identified proteins and the ordinate is the number of identified proteins, Figure S6: Proteins number distribution with GO in liver (A) and brain (B), Figure S7: Proteins number distribution with COG in liver (A) and brain (B), Figure S8: Proteins number distribution with KEGG in liver (A) and brain (B), Figure S9: Proteins number distribution with IPR in liver (A) and brain (B), Figure S10: The statistical results of subcellular localization of quantified proteins in liver (A) and brain (B), Figure S11: Real-time quantitative reverse-transcription PCR (qRT–PCR) expression analysis of differentially abundant proteins in liver (A) and brain (B). The changes in transcript and protein numbers are shown as log2 fold change values in pairwise comparisons. All qRT–PCR reactions were carried out in three biological replicates and three technical replicates (total of nine qRT–PCR reactions for each gene). ck_0h: control group; hy_5h: hypoxia group; re_5h: reoxygenation group, Figure S12: Focal adhesion (A) and ECM–receptor interaction (B) pathways in liver. Proteins in the green box indicate the DAPs involved in cytoskeleton organization, Table S1: All information of the indicators, Table S2: The primer sequences of DAPs used for qRT–PCR, Table S3: All quantified proteins in liver and brain of *Hypophthalmichthys molitrix*, Table S4: GO annotation, COG annotation, KEGG annotation, IPR annotation, and subcellular localization annotation of total quantified proteins in liver, Table S5: GO annotation, COG annotation, KEGG annotation, IPR annotation, and subcellular localization annotation of total quantified proteins in brain, Table S6: The differentially abundant proteins (DAPs) in liver among three groups, Table S7: The differentially abundant proteins (DAPs) in brain among three groups, Table S8: The distribution of liver differentially abundant proteins (DAPs) in Venn diagram, Table S9: The distribution of brain differentially abundant proteins (DAPs) in Venn diagram, Table S10: Liver differentially abundant proteins (DAPs) involved in cytoskeletal organization, Table S11: Measurement of intracellular GSH content and GSH/GSSG ratio in liver and brain.
