# Supplementary material for: Tandem Mass Tagging-Based Quantitative Proteomics Analysis Reveals Damage to the Liver and Brain of Hypophthalmichthys molitrix Exposed to Acute Hypoxia and Reoxygenation
Source: Antioxidants (Basel). 2022 Mar 19;11(3):589. doi: 10.3390/antiox11030589 (PMC8945220; doi:10.3390/antiox11030589)
Supplement: Supplementary file 1 [file antioxidants-11-00589-s001.zip › Figure S1.pdf]

A

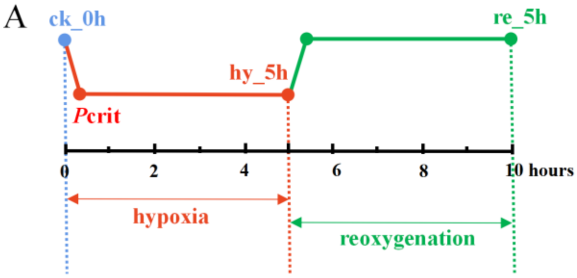

B

	Liver	Brain
Total spectra	430081	424731
Matched spectrum	114829	124776
Peptide	62273	72499
Identified protein	6802	8358
All	6794	8353

C

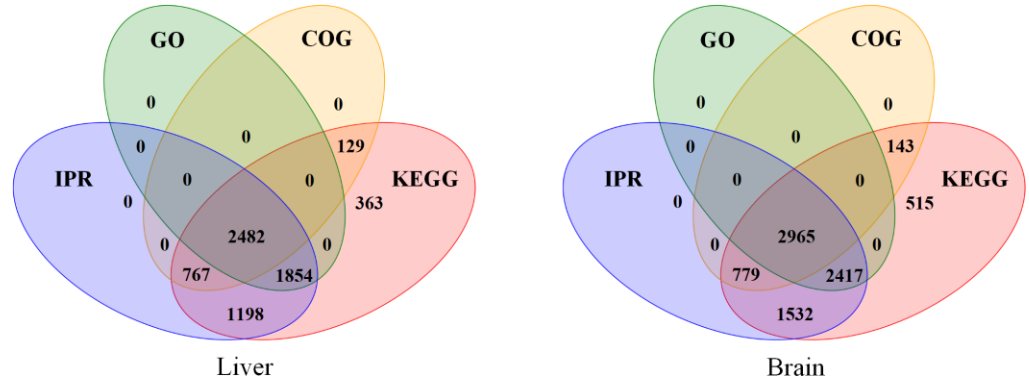

Figure S1. Analysis of the proteome in the liver and brain. (A) A schematic diagram of the experimental procedure. *P*crit, The critical oxygen tension of the experimental fish. (B) The total spectra, peptides, and proteins identified in the liver and brain, respectively. (C) Venn diagram of the statistics of the functional annotation in the liver and brain.
