# Supplementary material for: Tandem Mass Tagging-Based Quantitative Proteomics Analysis Reveals Damage to the Liver and Brain of Hypophthalmichthys molitrix Exposed to Acute Hypoxia and Reoxygenation
Source: Antioxidants (Basel). 2022 Mar 19;11(3):589. doi: 10.3390/antiox11030589 (PMC8945220; doi:10.3390/antiox11030589)
Supplement: Supplementary file 1 [file antioxidants-11-00589-s001.zip › Figure S11.pdf]

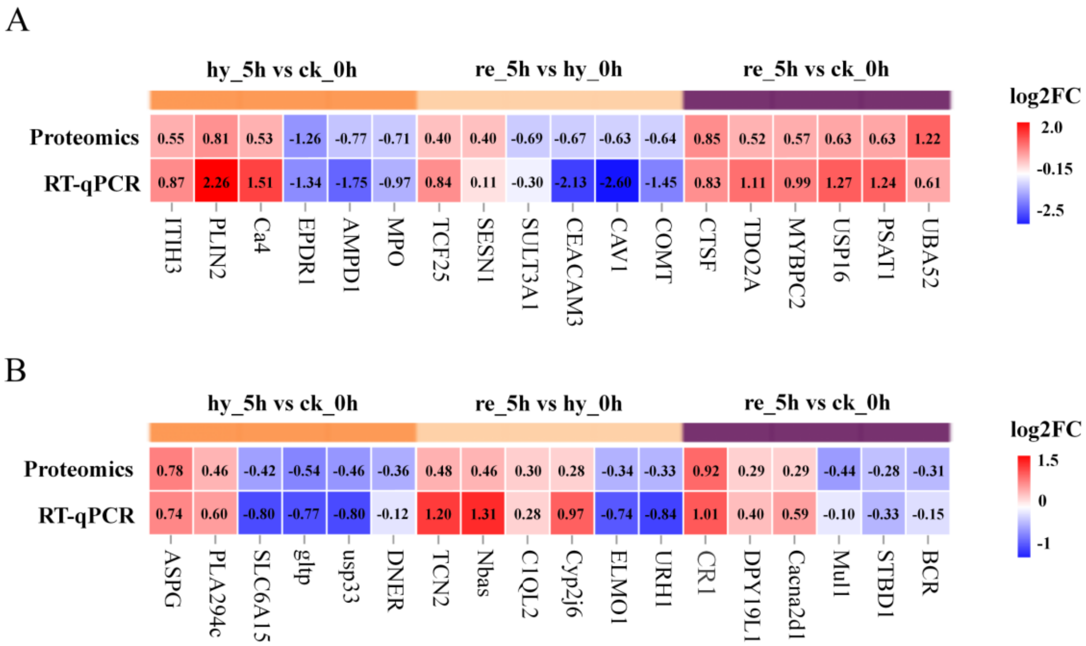

Figure S11. Real-time quantitative reverse-transcription PCR (RT-qPCR) expression analysis of differentially abundant proteins in liver (A) and brain (B). The changes in transcript and protein numbers are shown as log2 fold change values in pairwise comparisons. All RT-qPCR reactions were carried out in three biological replicates and three technical replicates (total of nine RT-qPCR reactions for each gene). ck_0h: control group; hy_5h: Hypoxia group; re_5h: Reoxygenation group.
