# Supplementary material for: Tandem Mass Tagging-Based Quantitative Proteomics Analysis Reveals Damage to the Liver and Brain of Hypophthalmichthys molitrix Exposed to Acute Hypoxia and Reoxygenation
Source: Antioxidants (Basel). 2022 Mar 19;11(3):589. doi: 10.3390/antiox11030589 (PMC8945220; doi:10.3390/antiox11030589)
Supplement: Supplementary file 1 [file antioxidants-11-00589-s001.zip › Figure S12.pdf]

A

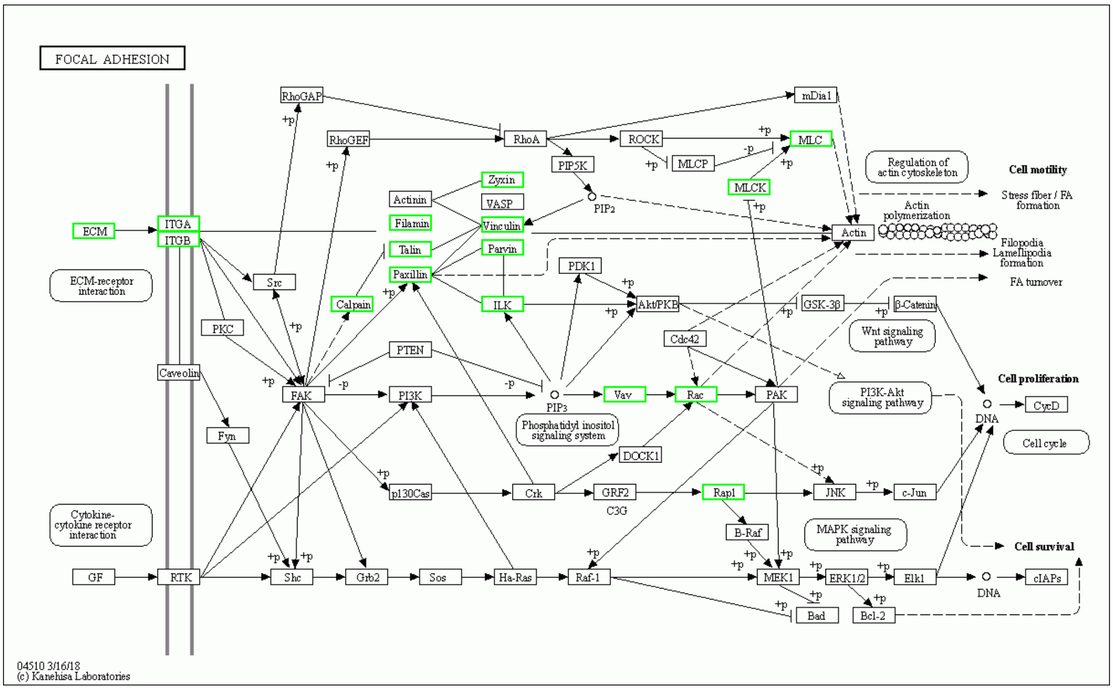

B

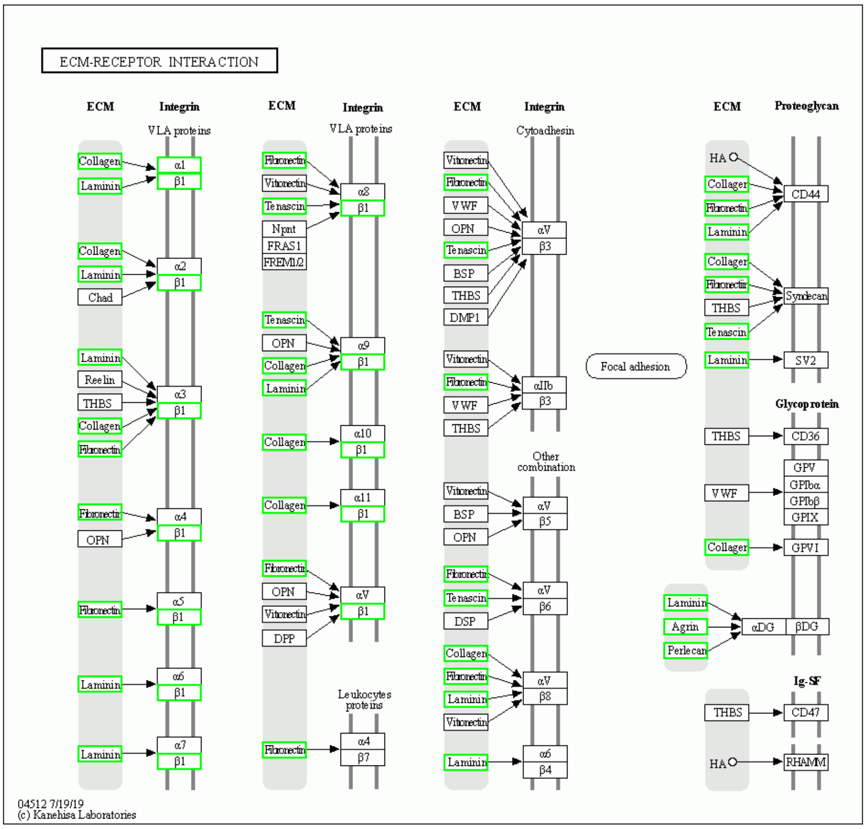

Figure S12. Focal adhesion (A) and ECM-receptor interaction (B) pathways in liver. Proteins in the green box indicate the DAPs involved in cytoskeleton organization.
