# Supplementary material for: Tandem Mass Tagging-Based Quantitative Proteomics Analysis Reveals Damage to the Liver and Brain of Hypophthalmichthys molitrix Exposed to Acute Hypoxia and Reoxygenation
Source: Antioxidants (Basel). 2022 Mar 19;11(3):589. doi: 10.3390/antiox11030589 (PMC8945220; doi:10.3390/antiox11030589)
Supplement: Supplementary file 1 [file antioxidants-11-00589-s001.zip › Figure S2.pdf]

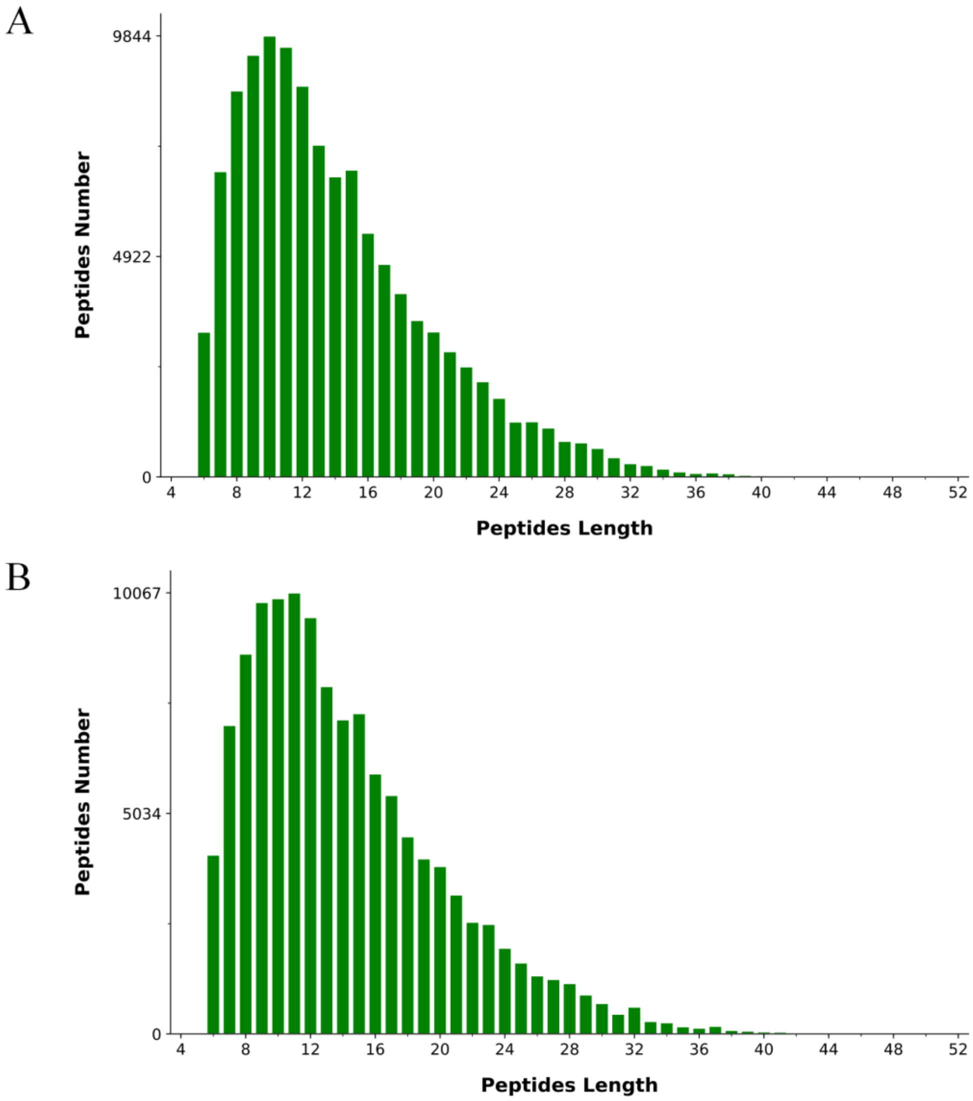

Figure S2. Peptide length distribution map of the identified peptides in liver (A) and brain (B). The abscissa indicates the amino acid residue number and the ordinate is the peptide number in the length of each peptide. The peptides length mainly distributed in 7-25.
