# Supplementary material for: Tandem Mass Tagging-Based Quantitative Proteomics Analysis Reveals Damage to the Liver and Brain of Hypophthalmichthys molitrix Exposed to Acute Hypoxia and Reoxygenation
Source: Antioxidants (Basel). 2022 Mar 19;11(3):589. doi: 10.3390/antiox11030589 (PMC8945220; doi:10.3390/antiox11030589)
Supplement: Supplementary file 1 [file antioxidants-11-00589-s001.zip › Figure S3.pdf]

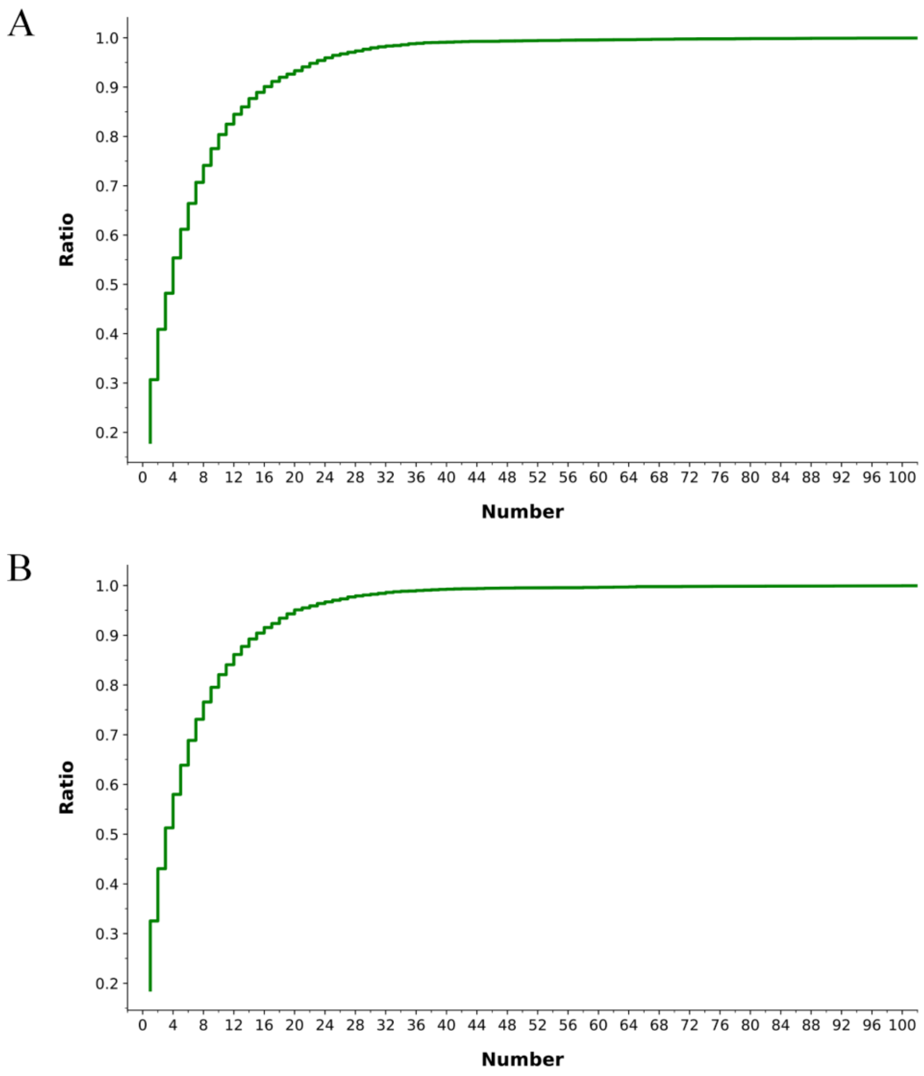

Figure S3. Unique peptide number distribution map of the identified peptides in liver (A) and brain (B). The abscissa indicates the number of Unique peptide and the ordinate is the ratio of protein contain Unique peptide to total protein.
