# Supplementary material for: Tandem Mass Tagging-Based Quantitative Proteomics Analysis Reveals Damage to the Liver and Brain of Hypophthalmichthys molitrix Exposed to Acute Hypoxia and Reoxygenation
Source: Antioxidants (Basel). 2022 Mar 19;11(3):589. doi: 10.3390/antiox11030589 (PMC8945220; doi:10.3390/antiox11030589)
Supplement: Supplementary file 1 [file antioxidants-11-00589-s001.zip › Figure S4.pdf]

A

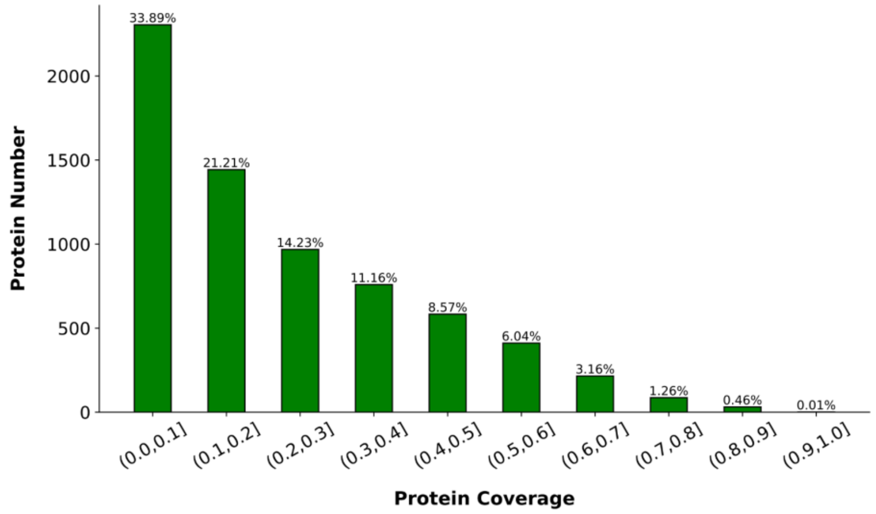

B

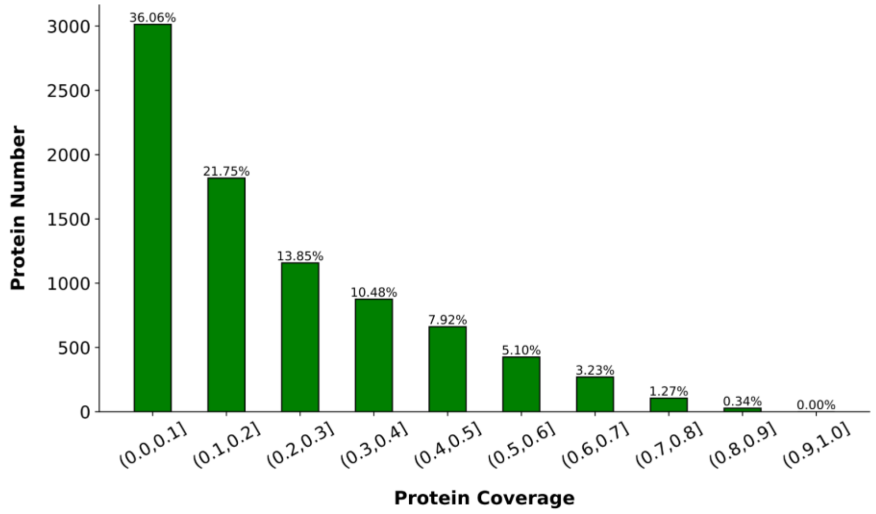

Figure S4. Protein coverage distribution map in liver (A) and brain (B). The abscissa indicates the protein coverage interval (the length of protein that covered by detected peptide/full-length the protein) and the ordinate is the protein number in each interval.
