# Supplementary material for: Tandem Mass Tagging-Based Quantitative Proteomics Analysis Reveals Damage to the Liver and Brain of Hypophthalmichthys molitrix Exposed to Acute Hypoxia and Reoxygenation
Source: Antioxidants (Basel). 2022 Mar 19;11(3):589. doi: 10.3390/antiox11030589 (PMC8945220; doi:10.3390/antiox11030589)
Supplement: Supplementary file 1 [file antioxidants-11-00589-s001.zip › Table S1.pdf]

Table S1 All information of the indicators

Sample	Indicator	Product Code
Serum	glutamic-pyruvic transaminase (ALT)	C009-3-1
Serum	glutamic-oxalacetic transaminase (AST)	C010-3-1
Serum	alkaline phosphatase (ALP)	A059-3-1
Serum	total protein (TP)	A045-3-2
Serum	albumin (ALB)	A028-1-1
Serum	globulin (GLB)	E026-1-1
Serum	total cholesterol (TC)	A111-1-1
Brain	acetylcholine (ACh)	A105-1-1
Brain	dopamine (DA)	H170

Note:. The indicators were measured using reagent kits and all kits were purchased at Nanjing Jiancheng Institute of Biotechnology, and the operation was according to the manufacturer's instructions.
