# Supplementary material for: Tandem Mass Tagging-Based Quantitative Proteomics Analysis Reveals Damage to the Liver and Brain of Hypophthalmichthys molitrix Exposed to Acute Hypoxia and Reoxygenation
Source: Antioxidants (Basel). 2022 Mar 19;11(3):589. doi: 10.3390/antiox11030589 (PMC8945220; doi:10.3390/antiox11030589)
Supplement: Supplementary file 1 [file antioxidants-11-00589-s001.zip › Table S11.pdf]

Table S11. Measurement of intracellular GSH content and GSH/GSSG ratio in liver and brain.

Sample	Indicator	Group	Value
Liver	Reduced glutathione (GSH) content (μmol/gport)	Control group	143.38 ± 9.21
		Hypoxia group	138.73 ± 7.43*
		Reoxygenation group	119.64 ± 5.25*
	Toal glutathione/Oxidized glutathione (T-GSH/GSSG)	Control group	47.61 ± 2.46
		Hypoxia group	34.31 ± 1.95*
		Reoxygenation group	27.64 ± 1.37**
Brain	Reduced glutathione (GSH) content (μmol/gport)	Control group	44.38 ± 2.36
		Hypoxia group	28.53 ± 1.94**
		Reoxygenation group	20.38 ± 1.85**
	Toal glutathione/Oxidized glutathione (T-GSH/GSSH)	Control group	35.84 ± 2.13
		Hypoxia group	27.01 ± 1.77*
		Reoxygenation group	22.67 ± 1.21**

Note: Values are expressed as the mean ± SD. The * $p < 0.05$ and ** $p < 0.01$ indicate significant differences compared with the control group. The indicators were measured using Reduced glutathione (GSH) assay kit (Product Code: A006-2-1), Toal glutathione/Oxidized glutathione assay kit (Product Code: A061-1-2). Both kits were purchased from Nanjing Jiancheng Institute of Biotechnology, and the operation was according to the manufacturer's instructions.
