# Supplementary material for: Tandem Mass Tagging-Based Quantitative Proteomics Analysis Reveals Damage to the Liver and Brain of Hypophthalmichthys molitrix Exposed to Acute Hypoxia and Reoxygenation
Source: Antioxidants (Basel). 2022 Mar 19;11(3):589. doi: 10.3390/antiox11030589 (PMC8945220; doi:10.3390/antiox11030589)
Supplement: Supplementary file 1 [file antioxidants-11-00589-s001.zip › Table S2.pdf]

Table S2 The primer sequences of DAPs used for RT-qPCR.

Group	Gene	Forward primer (5'-3')	Reverse primer (5'-3')
Liver hy_5h vs ck_0h	*ITIH3*	TGGTCAGTTCTACCGTGGATTG	GGGCACTTGTTCTCCGTTCTT
	PLIN2	GGTGAGGAGGAAGGATTCATAGT	TGGTCATTTGAGTCACAGTCGC
	Ca4	TGCTGGAGTCACAGGACATTCA	CCACACTCTCCGTTCACTTCAC
	Epdr1	GCAGGAGACATACACAAAGAACAG	GAGAGGTATTCAGCAGTCAGATGT
	AMPD1	GTGCGAGATTGCCAGGAACAGT	TGCGGATGTCGTTGCCCTCT
	Mpo	AGGCTGTGCGGCTCAAGTTC	GTGGGTTCCTCCTGTTGTTTGC
Liver re_5h vs hy_0h	*Tcf25*	CACACAAACAGCCCATAAGCAGAA	TCCACATCAGCCTCACCTATCTCT
	SESN1	GATGCTGAGGTGCCGTCTGT	CGCTCTCACACTCACACTAACAC
	SULT3A1	TGTTTAGTTGGCGGTTCATCTTGG	CGAGGCTGTGATTACCATGACCAT
	CEACAM3	CTGGAGGGACACTTACTGGAGAA	CCATCATGATCGAGCTCTCAACTGT
	CAV1	CCGCAGGAATACACTCACTCAC	GTCCACCTTCACCACATTGTCATT
	COMT	CGCCCTTTCTAATTTCCTCACCAT	GCAGAGAGCATATTGACCCAGTAG
Liver re_5h vs ck_0h	*Ctsf*	GGACCATTCTCAGCCAACCAAG	CTACACAGGACACAAGCAGAGC
	tdo2a	AGCAGCAGCAGCGATGACT	TGGTGATGGTAGGCTTGTGAGT
	Mybpc2	GAAGCAGCGTCAACAGCAGAGA	GAGCCGCCGTTAGTCATTCCAT
	usp16	AGCAGCGTCAGTGATGATGGA	CCGTCTCTGATTCTTGGCTTGTT
	PSAT1	GCCCAGCGAGTTTCTTTCCGAT	TTGTCCGAGGTGCCATGATTGTC
	UBA52	GCGTCTCAGAGGAGGCATCAT	GCGTAACACTTGCGGCAGAT
Brain hy_5h vs ck_0h	*ASPG*	TGTGGGTGTCCTGCGATTCTTC	CCGCTGCCGTAAGTCTCCAATA
	PLA2G4C	AAGAGGGTGGCTGGGAAATGGT	TCGCCTTCATCAGTCGGTGTCA
	SLC6A15	AGCGAGACAACAACTGCCACTT	GAAGCCCAGCACAGCGAACA
	gltp	CCTCATCTTCCTCCCTCCCAGT	CGCAGCACGGACCCTAATTGT

	usp33	GCAGTGGTACGAGTTCGATGAC	TCACCTTCCGCCTCTCCTTCT
	DNER	ACCTGCGATGTATGATGCGACTC	GGCTGTGTGTATGCGGTGTGT
	TCN2	AACCTACCCAATCCCAGTGTGC	GCCGACCACCAACTCACCATT
	nbas	ACACCCGTAAGACCTTCGTTT	CATCAAGGCTGAACCTCTGTAGTC
Brain	*C1QL2*	AGCAGCAGGTAGATGAAGGACTC	TGGCGGTCAGTTCTCTCAGTG
re_5h vs hy_0h	*Cyp2j6*	GACCACCTCCACCACTCTGTACTG	ACCCTCCCACAACACGATCAATCT
	ELMO1	GGATGGCTGGCTCTGGATAACA	CAATGCTACTGCGACCGAATGG
	URH1	CACAGACAAGGCTCGCTTTATG	ATGAATCCCTGCCCATCCACCA
	CFH	CAACAACCACGACGACCACAA	ACACCTCCTACACTCAATCCAACT
	DPY19L1	TCTACGCTCCTGTTGTTCCTCT	CCTGTCCTTCTCGCTGCTCT
Brain	*Cacna2d1*	AATCTGGTGCTTATTGTTGCTGAC	CCGTGGGTTCTGGGCTATCT
re_5h vs ck_0h	*mul1*	GCCTGTGCCACGCTATTCTACC	AGACACATCATCCTCCGCCAGA
	STBD1	AAGAGGACGAAGAACTGGCGAATA	GCTGGCTAGGAGAGTATGTGATGT
	BCR	CCTTGAAGCCTGACCGATTG	CACAGACAGGGGACTTTTCTTTTC
	β-actin	GAACCCCAAAGCCAACAG	CAGAGTCCATCACGATACCAG
