# Supplementary figures and images for: Tandem Mass Tagging-Based Quantitative Proteomics Analysis Reveals Damage to the Liver and Brain of Hypophthalmichthys molitrix Exposed to Acute Hypoxia and Reoxygenation
Source: Antioxidants (Basel). 2022 Mar 19;11(3):589. doi: 10.3390/antiox11030589 (PMC8945220; doi:10.3390/antiox11030589)

A

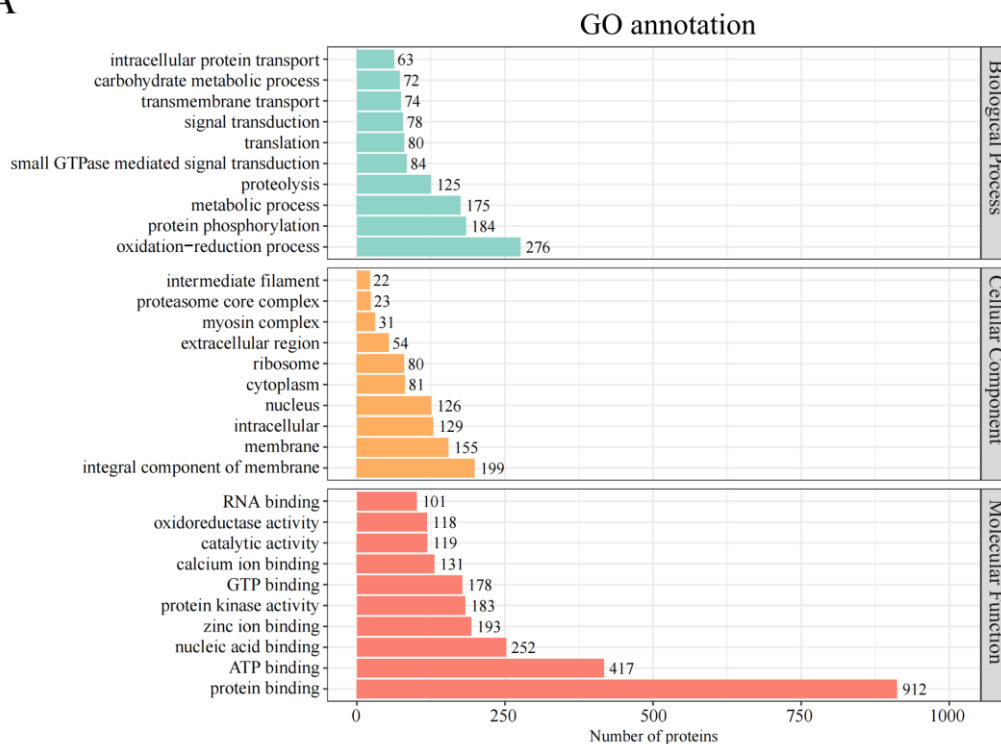

B

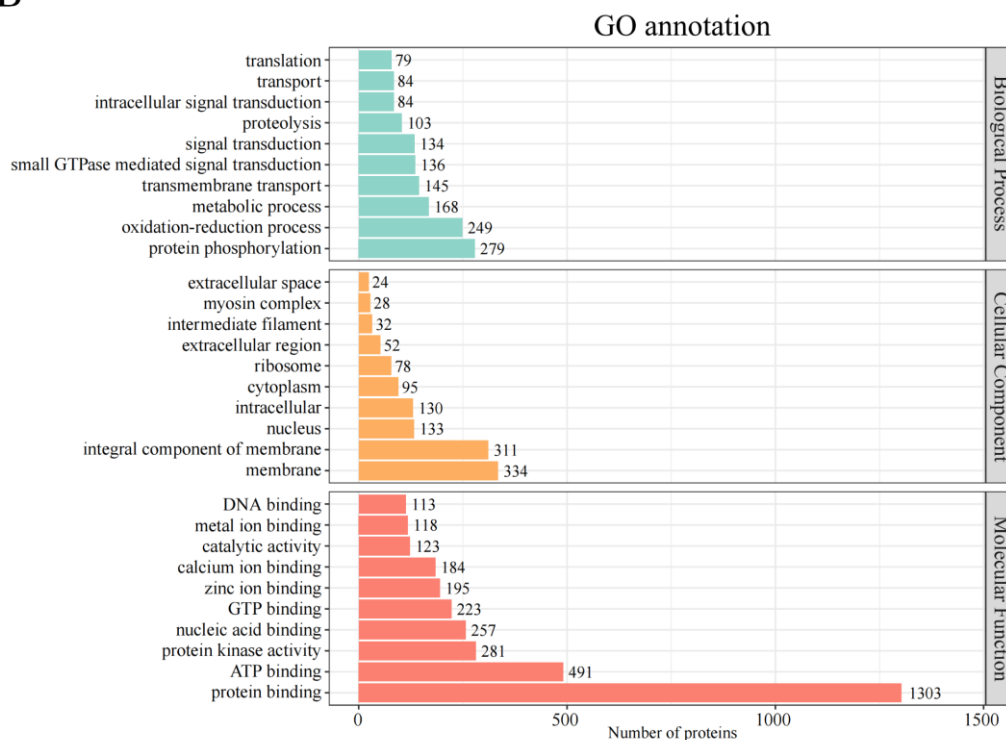

Figure S6. Proteins number distribution with GO in liver (A) and brain (B).

Supplement: Supplementary file 1 [file antioxidants-11-00589-s001.zip › Figure S6.pdf]

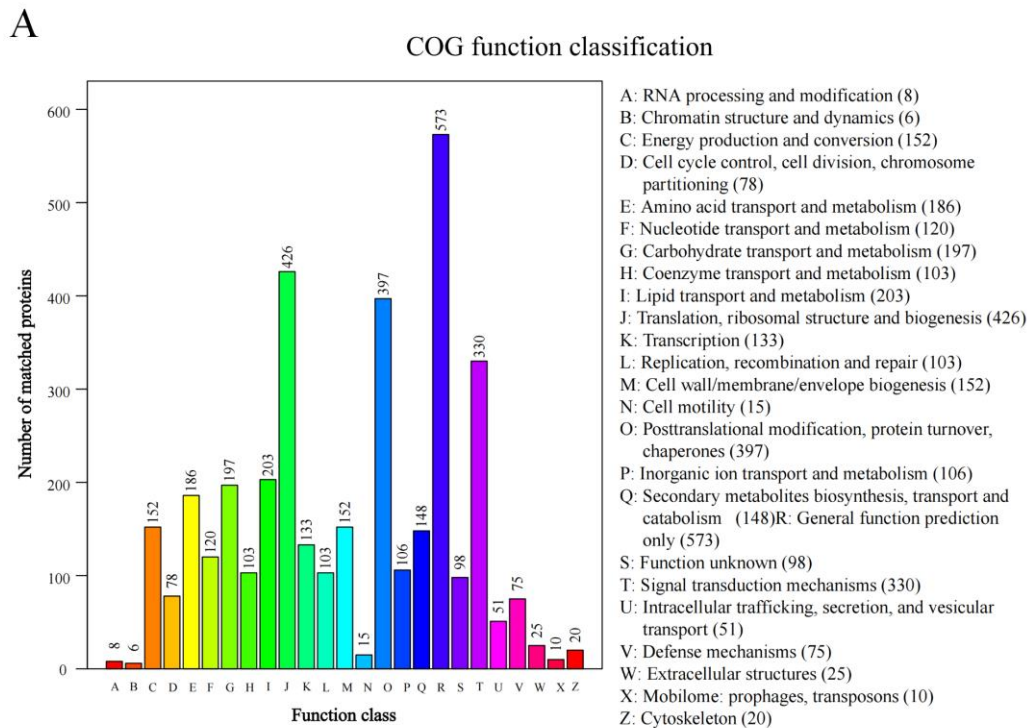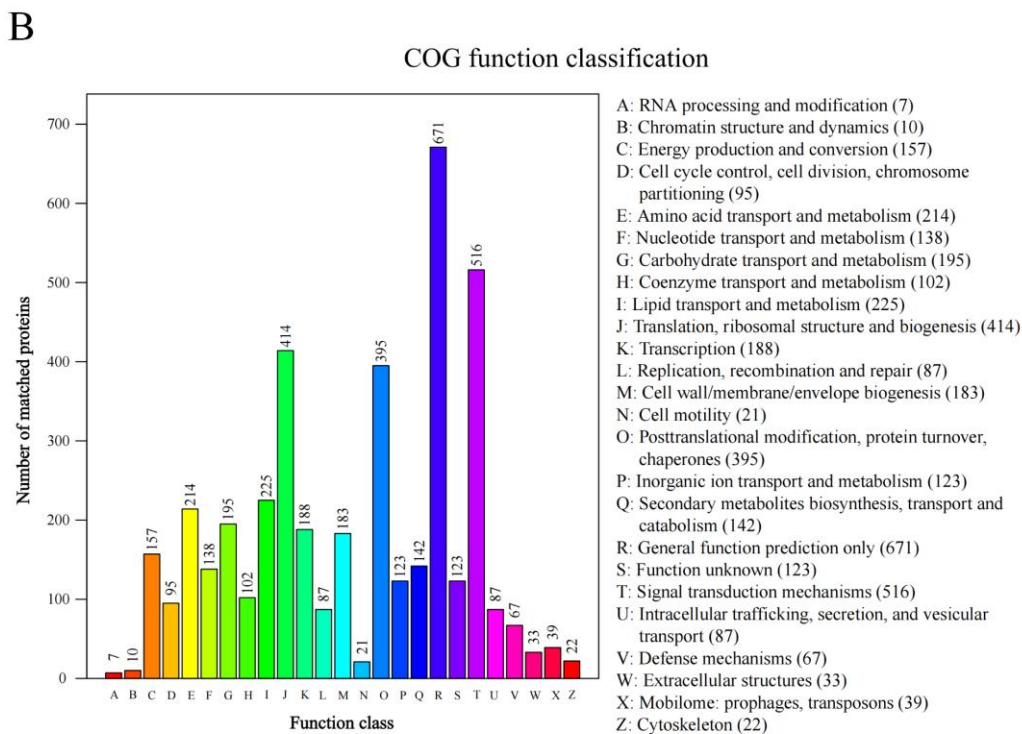

Figure S7. Proteins number distribution with COG in liver (A) and brain (B).

Supplement: Supplementary file 1 [file antioxidants-11-00589-s001.zip › Figure S7.pdf]

A

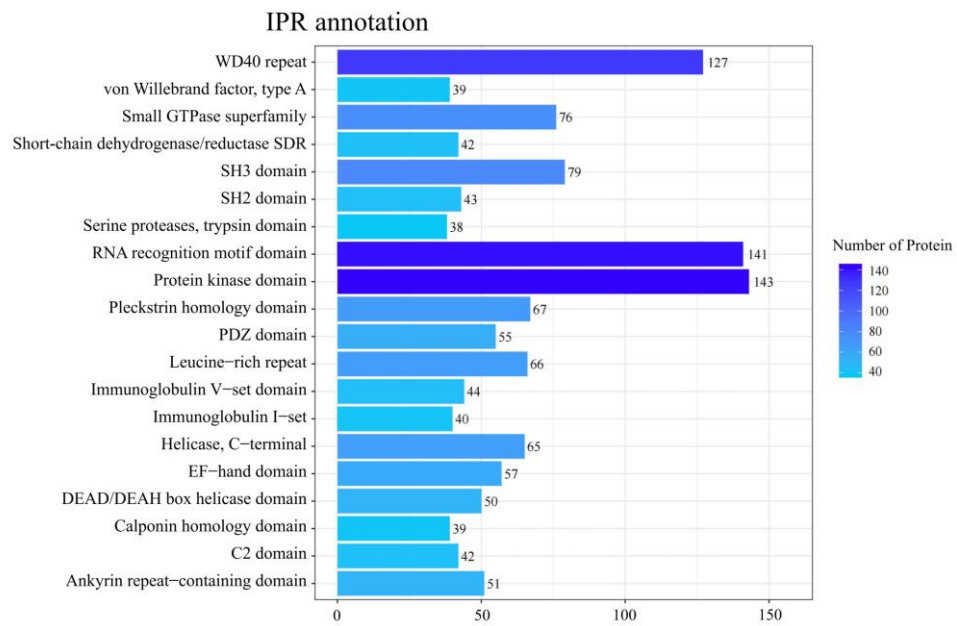

B

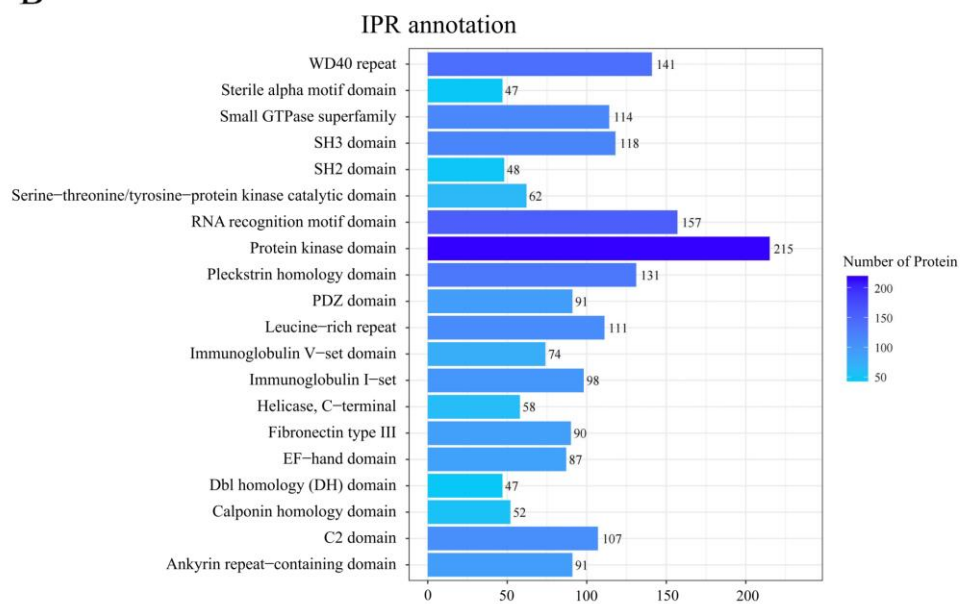

Figure S9. Proteins number distribution with IPR in liver (A) and brain (B).

Supplement: Supplementary file 1 [file antioxidants-11-00589-s001.zip › Figure S9.pdf]
